# Hypoxaemia and mortality in children with lower respiratory infection in low-income and middle-income countries: systematic review and meta-analysis

**DOI:** 10.1016/j.langlo.2026.104031

**Published:** 2026-09-01

**Authors:** Nadia E Hoekstra, Kristen S Joseph, Holly B Schuh, Laura Ruegsegger, Claire Twose, Tim Colbourn, Shubhada Hooli, William Checkley, Carina King, Stephen RC Howie, Hamish R Graham, Marzia Lazzerini, Tisungane Mvalo, Heather J Zar, Eric D McCollum

**Affiliations:** a Department of Pediatrics, University of North Carolina School of Medicine, Chapel Hill, NC, USA; b Global Program in Pediatric Respiratory Sciences, Eudowood Division of Pediatric Respiratory Sciences, Johns Hopkins University School of Medicine, Baltimore, MD, USA; c Department of Anesthesia and Critical Care Medicine, Johns Hopkins University School of Medicine, Baltimore, MD, USA; d Department of Epidemiology, Johns Hopkins Bloomberg School of Public Health, Baltimore, MD, USA; e Cincinnati Children’s Hospital Medical Center, Cincinnati, OH, USA; f Welch Medical Library, Johns Hopkins University School of Medicine, Baltimore, MD, USA; g Institute for Global Health, University College London, London, UK; h Division of Emergency Medicine, Department of Pediatrics, Baylor College of Medicine, Houston, TX, USA; i Division of Pulmonary and Critical Care, School of Medicine, Johns Hopkins University, Baltimore, MD, USA; j Center for Global Non-Communicable Disease Research and Training, Johns Hopkins University School of Medicine, Baltimore, MD, USA; k Department of Global Public Health, Karolinska Institutet, Stockholm, Sweden; l Department of Pediatrics, Child & Youth Health, University of Auckland, Auckland, Aotearoa New Zealand; m College of Medicine, Nursing and Health Sciences, Fiji National University, Suva, Fiji; n Melbourne Children’s Global Health, MCRI, University of Melbourne, Royal Children’s Hospital, Parkville, VIC, Australia; o Department of Paediatrics, University College Hospital Ibadan, Ibadan, Nigeria; p WHO Collaborating Centre for Maternal and Child Health, Institute for Maternal and Child Health IRCCS Burlo Garofolo, Trieste, Italy; q Department of Infectious Disease Epidemiology and International Health, London School of Hygiene & Tropical Medicine, London, UK; r University of North Carolina Project Malawi, Lilongwe, Malawi; s Department of Paediatrics & Child Health, Red Cross War Memorial Children’s Hospital, University of Cape Town, Cape Town, South Africa; t SA-MRC Unit on Child & Adolescent Health, University of Cape Town, Cape Town, South Africa; u Department of International Health, Johns Hopkins Bloomberg School of Public Health, Baltimore, MD, USA

## Abstract

**Background:**

Hypoxaemic lower respiratory infections (LRIs) are a leading cause of childhood mortality, with the highest burden in low-income and middle-income countries (LMICs). Hypoxaemia—low peripheral capillary oxyhaemoglobin saturation (SpO_2_)—is a marker of severity, and WHO recommends hospitalisation and oxygen administration for patients with SpO_2_ <90%. We aimed to update estimates from a 2015 systematic review and meta-analysis examining the association between hypoxaemia and mortality among children with LRIs in LMICs by incorporating studies published over the subsequent decade and evaluating mortality risk across multiple SpO_2_ thresholds.

**Methods:**

We conducted a systematic review with meta-analysis by searching PubMed, Embase, LILACS, Global Index Medicus, Web of Science, and Scopus for peer-reviewed studies published between Jan 1, 2015, and June 18, 2025, with combined terms related to pneumonia, children, mortality, and LMICs. We also included selected earlier studies through citation checking. Eligible studies reported associations between hypoxaemia and mortality in children younger than 5 years with LRIs in LMICs. We excluded case reports and case series with fewer than five deaths, studies focused exclusively on the neonatal period, and those limited to children with specific comorbidities or to postoperative patients, for consistency with the original review. Two reviewers independently screened studies, extracted data, and assessed quality. Eligible studies were combined with those from the original review and analysed using random-effects models to estimate odds ratios (ORs) by hypoxaemia threshold subgroup. The protocol was registered on PROSPERO (CRD42023433946).

**Findings:**

We identified 7734 records; 26 new studies met inclusion criteria and were combined with 18 from the original review. The 44 studies were published between 1993 and 2024 and were primarily from Africa (25 [57%] of 44) or Asia (19 [43%]); some studies spanned multiple locations. Data from 33 studies including 155 633 participants were included in the primary meta-analysis. Hypoxaemia of any threshold was associated with higher odds of LRI mortality (OR 4·36 [95% CI 3·52–5·39]) compared with no hypoxaemia. For SpO_2_ <90% versus 90–100%, OR for death was 4·75 (95% CI 3·42–6·58). For SpO_2_ 90–94% versus 95–100%, mortality risk was more than twice as high (OR 2·27 [95% CI 1·22–4·25]). Heterogeneity was substantial (*I*^2^ 64–85% across analyses), and eight (24%) of 33 studies in the primary meta-analysis had a high overall risk of bias; however, a sensitivity analysis restricted to studies with low or moderate risk of bias yielded similar results.

**Interpretation:**

SpO_2_ <90% strongly predicts mortality in children with LRIs in LMICs. Children with SpO_2_ 90–94% also have elevated risk, suggesting that paediatric LRI and pneumonia treatment algorithms should consider management at this hypoxaemia threshold.

**Funding:**

None.

## Introduction

Lower respiratory infections (LRIs), including pneumonia, remain the leading infectious cause of death in children younger than 5 years globally,^1^^,^^2^ despite the widespread use of *Haemophilus influenzae* type b (Hib) vaccines and pneumococcal conjugate vaccines (PCVs). The highest mortality rates occur in low-income and middle-income countries (LMICs), particularly in south Asia and Africa.

**Research in context**

**Evidence before this study**

Two previous systematic reviews have examined the association between hypoxaemia and mortality in children with lower respiratory infection (LRI) in low-income and middle-income countries (LMICs). In 2015, Lazzerini and colleagues reviewed 18 studies including 20 224 children, analysed 12 studies, and showed substantially increased odds of death among 13 936 children with LRI and peripheral capillary oxyhaemoglobin saturation (SpO_2_) <90% versus SpO_2_ 90–100% (odds ratio [OR] 5·47 [95% CI 3·93–7·63]). The authors also showed a similar hypoxaemia–mortality association among a subset of three studies including 673 children that compared SpO_2_ <92% with SpO_2_ 92–100% (3·66 [1·42–9·47]). This systematic review and meta-analysis was conducted largely before widespread implementation of *Haemophilus influenzae* type b (Hib) vaccines and pneumococcal conjugate vaccines (PCVs), which has since altered LRI epidemiology in many LMICs.

A more recent 2025 systematic review, by Graham and colleagues, focused primarily on hypoxaemia prevalence, and reported as a secondary finding that hypoxaemia was associated with higher odds of death compared with not having hypoxaemia (OR 4·56 [95% CI 2·97–7·00]) among 33 285 children admitted with primary respiratory conditions; however, most of the 23 included studies used a single hypoxaemia threshold of SpO_2_ <90%.

**Added value of this study**

Although previous work has established that hypoxaemia is common in LMICs and associated with mortality, existing evidence has largely focused on prevalence or on mortality risk using hypoxaemia defined in general terms or by a single threshold, without systematically evaluating mortality risk across multiple SpO_2_ thresholds. We provide updated, comprehensive, and significantly expanded estimates of the association between hypoxaemia and mortality in children with LRIs in LMICs across multiple SpO_2_ thresholds, including the WHO treatment threshold of SpO_2_ <90% and higher oxygen saturation ranges. We additionally examined mortality risk by altitude, LRI definition, and country-level Hib and PCV vaccination status, providing the most detailed stratified analysis to date.

**Implications of all the available evidence**

Hypoxaemia remains strongly associated with death among children with LRI in LMICs. Our findings show that children with SpO_2_ 90–94%, which is currently higher than the WHO referral and treatment threshold, also face meaningfully elevated mortality risk. Together with emerging evidence from outpatient and inpatient cohorts, these results support re-evaluation of hypoxaemia thresholds used to guide clinical management and referral decisions. Incorporating higher SpO_2_ thresholds into pneumonia and LRI management guidelines could help identify children at high risk earlier and reduce preventable deaths.

Children with LRIs who have low blood oxygen (ie, hypoxaemia) have a higher risk of death than those without hypoxaemia.^3^ Hypoxaemia is also common among children hospitalised with pneumonia in LMICs, with prevalence estimates ranging from 8% to 41% depending on the severity and the setting.^4^ Hypoxaemia is typically assessed by use of a pulse oximeter, a non-invasive device that measures peripheral capillary oxyhaemoglobin saturation (SpO_2_), which provides an estimate of the percentage of haemoglobin bound to oxygen in arterial blood. In healthy children at sea level, SpO_2_ values on room air typically approach 100%, although no single threshold for normal has been agreed upon. The most widely used guidelines for outpatient pneumonia management in LMICs are the WHO Integrated Management of Childhood Illnesses (IMCI) guidelines.^5^ The 2014 IMCI guidelines introduced outpatient screening for hypoxaemia when a pulse oximeter is available and recommend referral to hospital for children with SpO_2_ <90%. The WHO 2013 Pocket Book for Hospital Care for Children advises hospitalisation and oxygen therapy for children with hypoxaemia identified in hospital settings.^6^ Strong evidence supports that SpO_2_ <90% is associated with increased mortality in children with LRIs in LMICs.^3^^,^^7^

Emerging evidence shows that children with SpO_2_ <93% or <94% are also at elevated risk of death compared with children with SpO_2_ ≥93% or ≥94%.^8^^,^^9^ A 2015 systematic review and meta-analysis evaluated the association between hypoxaemia and mortality among children with LRIs in LMICs, reporting increased odds of death with SpO_2_ <90% compared with SpO_2_ 90–100% (odds ratio [OR] 5·47 [95% CI 3·93–7·63]) and SpO_2_ <92% compared with SpO_2_ 92–100% (3·66 [1·42–9·47]).^3^ Since that review, widespread implementation of Hib vaccines and PCVs has occurred, with Hib introduced in nearly all countries and PCV introduced in more than 150 countries, reaching global coverage of approximately 72–75% for Hib vaccination and 55–60% for PCV vaccination.^10^^,^^11^ This coverage has substantially reduced bacterial pneumonia incidence globally, including in LMICs with high LRI burden, and has shifted childhood LRI aetiology towards viral or viral-predominant causes with occasional bacterial co-infection.^12^ Studies of fatal childhood pneumonia, representing a distinct and severe clinical subset, show high rates of bacterial and polymicrobial infection.^13^ How this changing epidemiological landscape has influenced both the frequency of hypoxaemic LRI and the strength of its association with mortality is unclear.

We aimed to update estimates of the association between hypoxaemia and mortality among children with LRIs in LMICs and evaluate variation in risk across different hypoxaemia thresholds.

## Methods

### Search strategy and selection criteria

We conducted a systematic review and meta-analysis following the PRISMA guidelines.^14^

Search strategies were based on the original search by Lazzerini and colleagues^3^ and refined by a health sciences librarian (author CT). We searched PubMed, Embase, LILACS, Global Index Medicus, Web of Science, and Scopus for peer-reviewed studies published between Jan 1, 2015, and June 18, 2025, with combined terms related to pneumonia, children, mortality, and LMICs (eg, “pneumonia” AND “children” AND “risk factors” AND “mortality” AND “LMIC”; the full search strategy is in appendix 1 pp 2–17). We applied no language restrictions. In addition, we included studies published before 2015 that were identified through citation checking.

We included all peer-reviewed studies reporting the association between hypoxaemia and mortality in children younger than 5 years with LRI, and excluded case reports and case series with fewer than five deaths to maintain consistency with the original review.^3^ We expanded eligibility to include randomised controlled trials (RCTs) because of multiple seminal RCTs that were known to have relevant data related to hypoxaemia and mortality. For eligible RCTs, data from all randomised groups were included in analyses, consistent with standard meta-analytic practice. Countries were classified as LMICs using the World Bank system.^15^ We excluded studies focused exclusively on the neonatal period (<1 month of age) and those limited to children with specific comorbidities or to postoperative patients, consistent with the original review. Studies including children older than 5 years were eligible if 80% or more of the participants were younger than 5 years.

For the meta-analysis, we excluded studies from the original systematic review that did not provide mortality data for children with hypoxaemia and studies that reported SpO_2_ thresholds that were incompatible with our predefined SpO_2_ categories.

Two of three independent reviewers (KSJ, NEH, and LR) screened titles and abstracts; disagreements were resolved by the third reviewer. Full-text review was performed by two reviewers, with discrepancies resolved by consensus.

All data were extracted with Covidence, an online platform that supports systematic review workflows. We collected study characteristics (eg, country, setting, altitude, study period, LRI definition, hypoxaemia threshold, population characteristics, study design, sample size, type of analysis, and confounders), hypoxaemia prevalence, mortality numbers, and effect estimates (unadjusted and adjusted odds ratios [ORs], risk ratios [RRs], or hazard ratios [HRs], along with their 95% CIs). In terms of comorbidities, we documented severe acute malnutrition, HIV infection, and diarrhoea. We also recorded country-level Hib and PCV vaccine status using International Vaccine Access Center data^16^ and documented whether each vaccine was included in the national immunisation programme before or during the study period.

The protocol was registered on PROSPERO (CRD42023433946).

### Data analysis

Two reviewers (NEH and KSJ) independently assessed the risk of bias of each study using the Quality in Prognosis Studies tool, applying the approach from the original review (appendix 1 pp 18–20).^3^ Six domains were evaluated: study participation, study attrition, prognostic factor measurement, outcome measurement, study confounding, and statistical analysis and reporting. Each domain was graded as low, moderate, high, or unknown risk of bias. Discrepancies were resolved by consensus. We also applied the original review’s overall risk of bias rating system, classifying each study as low, moderate, or high (appendix 1 p 21).

All statistical analyses were conducted with Stata 17 SE.

For the systematic review, we summarised study-level characteristics and calculated hypoxaemia prevalence, overall mortality, and mortality among children with hypoxaemia when these measures were not reported by the study but could be derived from reported data. We combined extracted data from newly included studies with data from studies included in the original systematic review. When possible, we used data reported by Lazzerini and colleagues;^3^ however, we re-reviewed each original study to assure accuracy and extracted additional data when available. We excluded study participants with missing or failed SpO_2_ measurements when possible. We sought additional data from study authors when feasible; however, studies without mortality data for predefined hypoxaemia thresholds were excluded from threshold analyses.

For the meta-analysis, we prioritised the use of adjusted ORs, followed by unadjusted ORs either reported or calculated from descriptive data. If RRs were reported, we assessed the rarity of death in the study population and prioritised adjusted RRs, followed by calculated unadjusted ORs when appropriate.

To account for interstudy heterogeneity, we implemented the restricted maximum likelihood random effects model to estimate pooled odds ratios for death, using a-priori defined hypoxaemia subgroups and the inverse-variance weighting method. Heterogeneity between studies was evaluated using forest plots, τ^2^, and *I*^2^ statistics. *I*^2^ values of 25–50%, >50–75%, and >75% were interpreted as low, moderate, and high heterogeneity, respectively. We used τ^2^ to quantify the absolute magnitude of between-study variability in the underlying effect estimates, with larger values indicating greater variability in true effects across studies. Potential publication bias and small-study effects were evaluated using funnel plots, Begg and Egger tests, and leave-one-out analyses to identify studies to exclude from the primary analysis. Point estimates with 95% CIs not spanning the null value of 1·00 were considered statistically significant.

We defined hypoxaemia subgroups a priori for analysis. The SpO_2_ thresholds were categorised on the basis of the WHO treatment threshold: WHO hypoxaemia*,* defined as SpO_2_ <90% compared with ≥90%; above WHO hypoxaemia*,* which included studies with SpO_2_ thresholds of <95%, <94%, <93%, or <92%, each compared with children with SpO_2_ at or above the respective threshold; and below WHO hypoxaemia, defined as SpO_2_ <85%, compared with ≥85%. We also conducted a separate analysis of moderate hypoxaemia, defined as SpO_2_ 90–94% compared with 95–100%. For studies reporting multiple hypoxaemia thresholds, we selected the highest threshold (eg, using <95% rather than <90%) to ensure that the study could be included in the broadest relevant subgroup.

To quantify mortality burden, we estimated the population attributable fraction (PAF) for hypoxaemia using Miettinen’s formula, which estimates the proportion of deaths among children with LRI that could be attributable to hypoxaemia, assuming a causal relationship.^17^ PAFs were calculated for each predefined SpO_2_ threshold comparison, for hypoxaemia of any threshold (overall), and for moderate hypoxaemia (SpO_2_ 90–94%), by use of data from studies included in the primary meta-analysis and all LRI deaths as the outcome denominator.

Subgroup analyses were conducted to explore the effects of various factors on mortality risk. We examined the effect of altitude by categorising studies on the basis of the elevation of the study population’s location: 0–1499 metres, 1500–2499 metres, and ≥2500 metres above sea level. These categories were selected to maintain consistency with the original systematic review and with the altitude-adjusted SpO_2_ thresholds discussed in WHO oxygen therapy guidance.^18^

We also assessed different study definitions of LRI, categorised as WHO, enriched WHO, and other. The WHO definition refers to the WHO IMCI clinical criteria.^5^ Enriched WHO includes the WHO definition plus additional diagnostic criteria (eg, radiographic or laboratory findings). Definitions categorised as other do not incorporate WHO IMCI criteria and instead rely on local clinical guidelines, radiological criteria, or investigator-defined criteria. Each study’s LRI definition can be found in appendix 1 (pp 22–23).

Finally, we considered vaccination for each study country, categorised as neither PCV nor Hib (ie, neither PCV nor Hib vaccination was introduced); partial Hib (ie, Hib vaccination was introduced during the study period but without PCV); Hib (ie, Hib vaccination was available during the entire study period but PCV was not); partial PCV, partial Hib (ie, PCV and Hib vaccination were introduced during the study period); partial PCV, Hib (ie, PCV was introduced during the study period and Hib vaccination was available during the entire study period); and PCV and Hib (ie, PCV and Hib vaccination were available during the entire study period).

Sensitivity analyses were performed to evaluate the robustness of the pooled estimates. First, we examined the effect of removing influential studies by assessing changes in assigned weight, point estimates, and CIs on pooled ORs. We also assessed the effect of restricting analyses to unadjusted ORs that were either reported in the original manuscripts or calculated from available data (ie, total study population, hypoxaemic population, hypoxaemic deaths, and non-hypoxaemic deaths), without substituting other effect measures such as adjusted ORs or RRs. Additionally, we evaluated the effect of excluding studies judged to have high risk of bias.

As tests for small-study effects (Egger regression-based tests) indicated potential bias in the preliminary analysis, we excluded seven studies with 130 or fewer participants^19^, ^20^, ^21^, ^22^, ^23^, ^24^, ^25^ and two additional studies identified as contributing to small-study bias^26^^,^^27^ from the primary meta-analysis and all subsequent analyses. As a sensitivity analysis, we repeated the meta-analysis including all studies to assess the effect of these exclusions on pooled estimates.

Certainty of evidence for the primary meta-analysis outcomes was assessed by use of an adapted GRADE approach, applied with GRADEpro GDT 2015 software. Because hypoxaemia was evaluated as a prognostic exposure, all evidence was considered observational and initially rated as low certainty, with downgrading for study limitations, inconsistency, indirectness, imprecision, and publication bias, and upgrading for magnitude of effect and dose response where appropriate.

### Role of the funding source

There was no funding source for this study.

## Results

We screened 7734 unique articles and identified 81 for full text review. Of these, 26 studies met the inclusion criteria and were added to the 18 studies from the original review, resulting in a total of 44 studies (figure 1).^9^^,^^19^, ^20^, ^21^, ^22^, ^23^, ^24^, ^25^, ^26^, ^27^, ^28^, ^29^, ^30^, ^31^, ^32^, ^33^, ^34^, ^35^, ^36^, ^37^, ^38^, ^39^, ^40^, ^41^, ^42^, ^43^, ^44^, ^45^, ^46^, ^47^, ^48^, ^49^, ^50^, ^51^, ^52^, ^53^, ^54^, ^55^, ^56^, ^57^, ^58^, ^59^, ^60^, ^61^ Characteristics of studies are summarised in the table.

**Figure 1:**
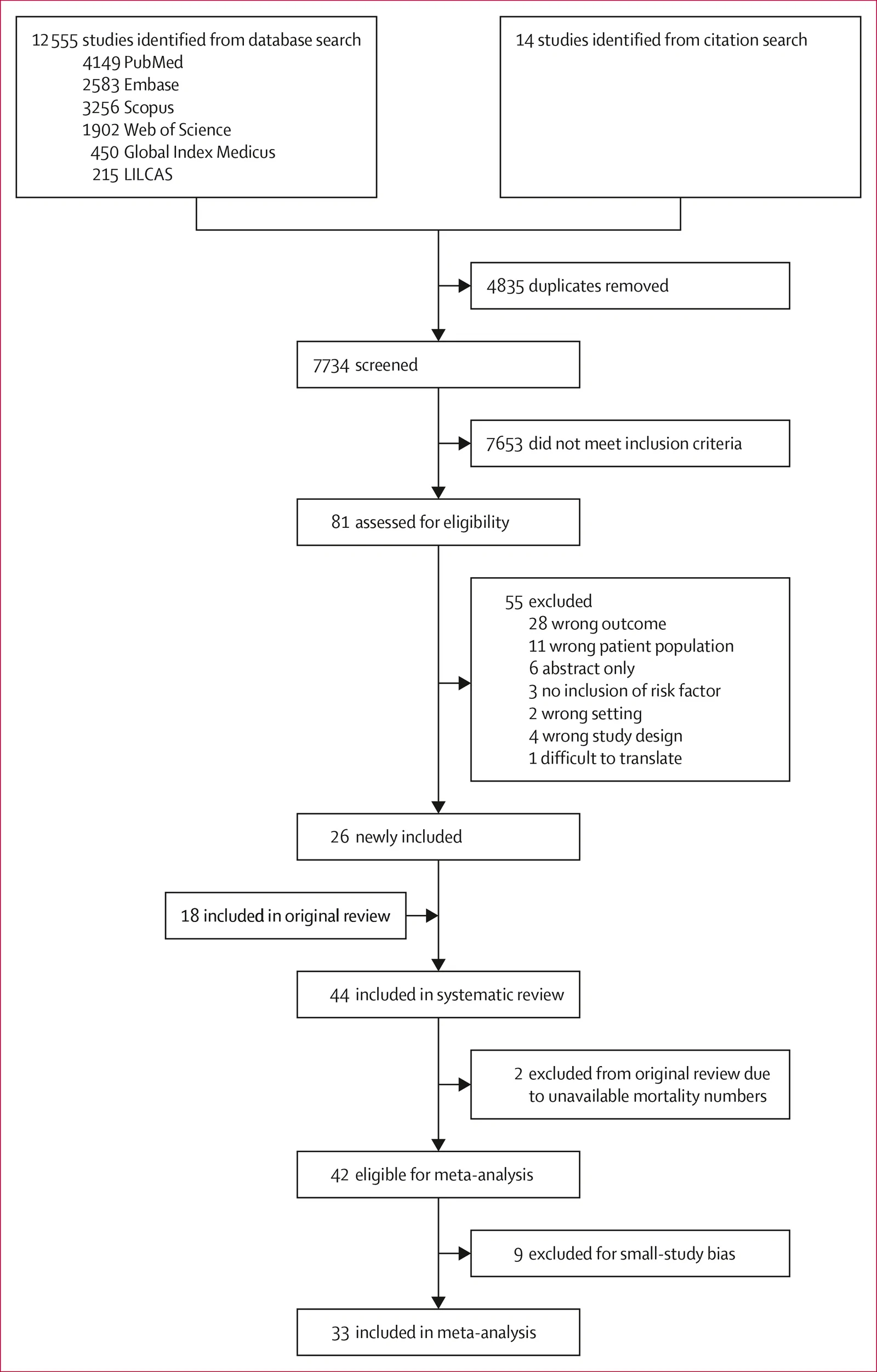
Study selection

**Table:** Characteristics of studies included in the systematic review

	SpO_2_ threshold	Country	Time period	Country vaccination	Age of population	Setting	Participants evaluated for hypoxaemia∗	LRI mortality rate	Hypoxaemia prevalence†
Abdulkadir et al (2015)^26^	<90%	Nigeria	2010–11	Neither PCV nor Hib	2–59 months	Urban hospital	200	8·5%	41·5%
Alwadhi et al (2017)^19^	<90%	India	2013–15	Hib	2–60 months	Tertiary hospital	112	3·6%	50·9%
Aroor et al (2022)^20^	<90%	India	2018	PCV and Hib	1–60 months	Urban tertiary hospital	28	25·0%	85·7%
Asghar et al (2008)^28^	Various (≤90% and ≤88% at high altitude sites)	Bangladesh, Ecuador, India, Mexico, Pakistan, Yemen, and Zambia	2020–24	PCV and Hib	2–59 months	Multiple: urban hospitals	958	6·8%	64·6%
Awasthi et al (2023)^29^	Various (93–100%, 90–92%, and <90%)	India	2015–22	Partial PCV, Hib	2–59 months	Multiple: district hospitals	6745	1·4%	14·5%
Bassat et al (2016)^30^	<90%	Mozambique	2006–07	Neither PCV nor Hib	0–59 months	Rural hospital	825	9·6%	27·9%
Bénet et al (2017)^31^	<90%	India, Madagascar, Mali, and Paraguay	2010–14	Partial PCV, Partial Hib	2–60 months	Multiple: tertiary hospitals	405	3·5%	17·3%
Bui-Binh-Bao et al (2021)^27^	<90%	Viet Nam	2019–20	Hib	2–60 months	Urban hospital	281	2·8%	11·4%
Chakraborty et al (2020)^32^∗∗	<90%	India	2016–17	Partial PCV, Hib	2–59 months	Urban hospital	285	33·3%	50·2%
Chisti et al (2011)^33^	<90%	Bangladesh	2007	Neither PCV nor Hib	0–59 months	Urban hospital	198	12·1%	NA
Chisti et al (2013)^34^	<90%	Bangladesh	2011–12	Hib	0–59 months	Urban hospital	140	25·0%	12·1%
Colbourn et al (2020)^35^‡‡	Various (≥93%, <93%, and <90%)	Malawi	2012–14	PCV and Hib	0–59 months	Multiple: health centres and rural community health worker settings	643	2·0%	10·1%
Dembele et al (2019)^36^	<90%	Philippines	2008–16	Partial Hib	2–59 months	Multiple: secondary and tertiary hospitals	4279	4·60%	13·2%
Demers et al (2000)^37^	Various (≥95%, 90–94%, 85–89%, and <85%)	Central African Republic	1996–97	Neither PCV nor Hib	0–59 months	Urban hospital	392	12·50%	16·8%
Dias et al (2024)^38^	<95%	Brazil	2020–23	PCV and Hib	<18 years	Multiple: urban and rural hospitals	194 863	3·0%	57·6%‡
Djelantik et al (2003)^39^	<85%	Indonesia	1999–2001	Neither PCV nor Hib	0–23 months	Multiple: rural hospitals	4306	11·6%	78·1%§
Duke et al (2001)^40^∗∗	Various (70–84%, 50–69%, and <50%)	Papua New Guinea	1998–99	Neither PCV nor Hib	1–59 months	Rural hospital	703	6·5%	46·5%¶
Graham et al (2011)^42^	<90%	Malawi	2005–06	Hib	2–156 months (12% >60 months)	Urban hospital	327	10·1%	36·4%
Graham et al (2019)^41^	<90%	Nigeria	2015–17	PCV and Hib	28 days to 15 years	Multiple: secondary hospitals	2073	7·2%	23·4%
Hooli et al (2020)^43^‡‡	Various (>92%, 90–92%, and <90%)	Malawi	2011–14	Partial PCV, Hib	0–2 months	Multiple: health centre and rural, district, and tertiary hospitals	1491	2·3%	14·7%
Junge et al (2006)^44^	Various (>94%, 90–94%, and<90%)	The Gambia	1993–94	Neither PCV nor Hib	41% <12 months	Urban hospital	456	4·4%	12·5%
Kapoor et al (2022)^45^	<90%	India	2017–18	Partial PCV, Hib	2–59 months	Urban hospital	180	9·4%	36·1%
Kuti et al (2013)^46^	<90%	The Gambia	2010–11	PCV and Hib	2–59 months	Rural hospital	389	3·8%	18·8%
McNally et al (2007)^48^	<92%	South Africa	2001–02	Hib	1–59 months	Urban hospital	358	15·1%	NA
McCollum et al (2019)^47^	Various (93–100%, 90–92%, and <90%)	Malawi	2015–18	PCV and Hib	1–59 months	Rural hospital	644	13·7%	64·4%
McCollum et al (2023)^9^‡‡	Various (94–100%, 90–93%, and <90%)	Bangladesh	2015–17	Partial PCV, Hib	3–35 months	Multiple: rural clinics	6951	0·3%	2·0%
Mwaniki et al (2009)^49^	<90%	Kenya	2002–05	Hib	0–59 months	Rural hospital	13 183	5·7%	5·3%
Nantanda et al (2008)^21^	<92%	Uganda	2005–06	Hib	2–59 months	Urban hospital	157	15·3%	39·5%||
Nantanda et al (2014)^50^	<92%	Uganda	2011–12	Hib	2–59 months	Urban hospital	614	3·6%	NA
Odeyemi et al (2021)^22^	<90%	Nigeria	2017	PCV and Hib	1–60 months	Tertiary hospital	129	10·1%	38·0%
Onyango et al (1993)^51^∗∗	<90%	Kenya	1989	Neither PCV nor Hib	0–36 months	Urban hospital	256	8·2%	59·0%
Pathak and Gupta (2020)^23^	<90%	India	2019	PCV and Hib	1–59 months	Urban hospital	75	14·7%	13·3%
Pranathi et al (2023)^24^	<90%	India	2022	PCV and Hib	2–12 months	Urban hospital	68	7·3%	39·7%
Rahman et al (2021)^52^	<90%	Bangladesh	2014–17	Partial PCV, Hib	2–59 months	Urban hospital	2646	6·4%	40·0%
Ramakrishna et al (2012)^53^	Various (>90%, 80–89%, and <80%)	Malawi	2005–06	Hib	2 months to 14 years	Urban hospital	233	10·7%	37·3%
Reed et al (2012)^54^∗∗	<90%	South Africa	1998–2001	Partial Hib	<24 months	Urban hospital	4148	7·2%	37·9%
Rodriguez et al (2010)^55^††	<90%	Colombia	2007–08	Hib	<60 months	Multiple: urban hospitals	226	NA	NA
Shi et al (2020)^25^	<90%	China	2013–17	Neither PCV nor Hib	1 month to 12 years	Urban hospital	113	12·4%	29·2%
Sigaúque et al (2009)^56^	<90%	Mozambique	2004–06	Neither PCV nor Hib	0–23 months	Rural hospital	584	13·0%	6·5%
Smyth et al (1998)^57^	<92%	Zambia	1994–95	Neither PCV nor Hib	1–59 months	Rural hospital	158	14·6%	34·8%||
Tekam et al (2023)^58^	Various (80–91% and <80%)	India	2021–22	PCV and Hib	2–60 months	Urban hospital	240	21·7%	80·8%||
Usen et al (1999)^59^	<90%	The Gambia	1993–95	Neither PCV nor Hib	2–33 months	Multiple: urban hospitals	1072	3·4%	5·9%
Zampoli et al (2017)^60^	<90%	South Africa	2011	PCV and Hib	0–13 years	Urban hospital	206	8·7%	70·9%
Zhang et al (2013)^61^	<90%	China	2007–10	Neither PCV nor Hib	0–60 months	Urban hospital	707	5·8%	45·0%

Vaccination for each study country was categorised as neither PCV nor Hib (ie, neither PCV nor Hib vaccination was introduced); partial Hib (ie, Hib vaccination was introduced during the study period but without PCV); Hib (ie, Hib vaccination was available during the entire study period but PCV was not); partial PCV, partial Hib (ie, PCV and Hib vaccination were introduced during the study period); partial PCV, Hib (ie, PCV was introduced during the study period and Hib vaccination was available during the entire study period); and PCV and Hib (ie, PCV and Hib vaccination were available during the entire study period). Hib=*Haemophilus influenzae* type b. LRI=lower respiratory infection. PCV=pneumococcal conjugate vaccine. SpO_2=_peripheral capillary oxyhaemoglobin saturation.

∗ Number of participants for whom hypoxaemia status was assessed or reported. Participants with missing, failed, or unreported oxygen saturation measurements were excluded from this count where identified.

† For SpO_2_ <90% unless otherwise specified.

‡ SpO_2_ <95%.

§ SpO_2_ <85%.

¶ SpO_2_ <70%.

|| SpO_2_ <92%.

∗∗ Study conducted at moderate altitude (1500–2499 metres above sea level).

†† Study conducted at high altitude (≥2500 metres above sea level).

‡‡ Study includes outpatient clinic or health centre data.

Included studies were published between 1993 and 2024. Of the 44 included studies, 25 (57%) involved sites in Africa and 19 (43%) involved sites in Asia; these categories are not mutually exclusive because some studies spanned multiple continents. Additionally, two studies were conducted exclusively in South America. 42 (95%) studies were at least partly hospital-based, with 32 (73%) conducted in urban or tertiary referral hospitals (table). Only one study, from Colombia, involved a population living 2500 metres or more above sea level (appendix 1 pp 22–23).^55^ 13 (30%) studies were conducted before national PCV or Hib vaccination implementation (table). 27 studies (61%) used the WHO definition of LRI, four (9%) used an enriched WHO definition, and 13 (30%) used other criteria (appendix 1 pp 22–23).

27 (61%) studies only evaluated the WHO treatment threshold of SpO_2_ <90%. 11 (25%) studies assessed multiple hypoxaemia thresholds. Hypoxaemia prevalence ranged from 2·0% (139 of 6951) to 85·7% (24 of 28) for studies with a threshold of SpO_2_ <90%, and mortality ranged from 0·3% (23 of 6951) to 33·3% (95 of 285). All studies reported SpO_2_ measurement at presentation or hospital admission when timing was specified, and measurements were obtained on room air when reported. In studies reporting serial measurements, mortality risk was generally evaluated with the presentation or admission SpO_2_.

For the meta-analysis, we excluded two studies from the original systematic review: one that had been excluded from the original meta-analysis because it did not provide mortality data for children with hypoxaemia, and one that was included but reported only SpO_2_ thresholds of less than 85% and provided no data for SpO_2_ measurements of 85% or higher, making it incompatible with our predefined SpO_2_ categories. Thus, 42 studies met criteria for inclusion in the meta-analysis, with 33 ultimately retained in the primary meta-analysis after exclusions for potential small-study or publication bias. The primary meta-analysis included 155 633 participants across the 33 studies. Hypoxaemia of any threshold, compared with no hypoxaemia, was associated with 4·36-times (95% CI 3·52–5·39) higher odds of death among children with LRI, corresponding to more than a four-times increase in mortality risk (figure 2). Substantial heterogeneity was observed (*I*^2^=85·21%), with variability in effect estimates across studies (τ^2^=0·27).

**Figure 2:**
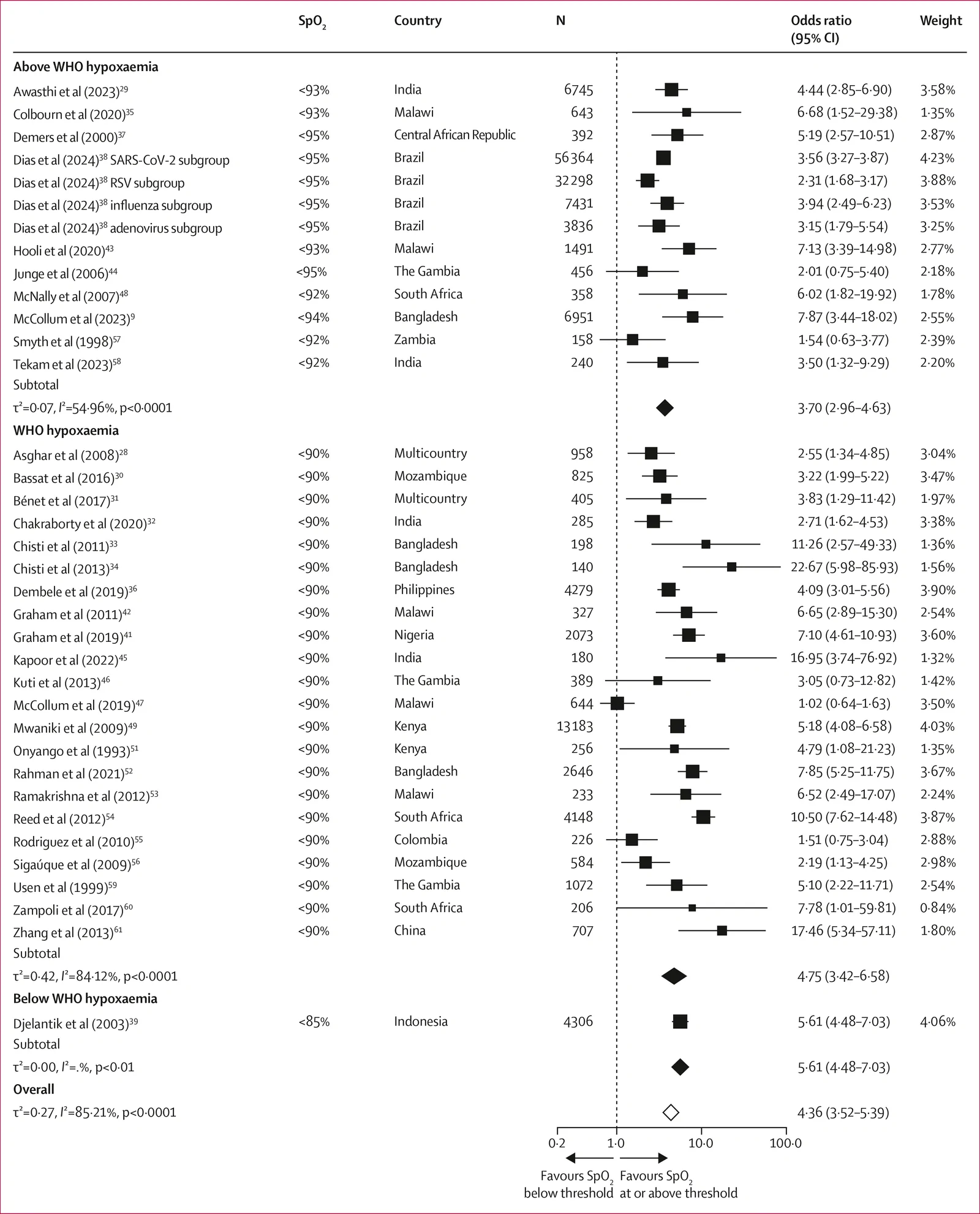
Odds of mortality among children with lower respiratory infection comparing oxygen saturation below versus at or above specified SpO_2_ thresholds, by threshold subgroup and overall For studies reporting multiple hypoxaemia thresholds, we selected the highest threshold to ensure that the study could be included in the broadest relevant subgroup. Analyses used the random-effects restricted maximum likelihood model. RSV=respiratory syncytial virus. SpO_2=_peripheral capillary oxyhaemoglobin saturation.

The OR of death from LRI for WHO-defined hypoxaemia (SpO_2_ <90%) compared with SpO_2_ 90–100% was 4·75 (95% CI 3·42–6·58), indicating more than four-times higher odds of death (figure 2). Heterogeneity was high (*I*^2^=84·12%). When restricted to SpO_2_ 90–94%, versus SpO_2_ 95–100%, the OR of death from LRI was 2·27 (95% CI 1·22–4·25), indicating more than double the odds of death (figure 3). Heterogeneity was moderate (*I*^2^=63·84%).

**Figure 3:**
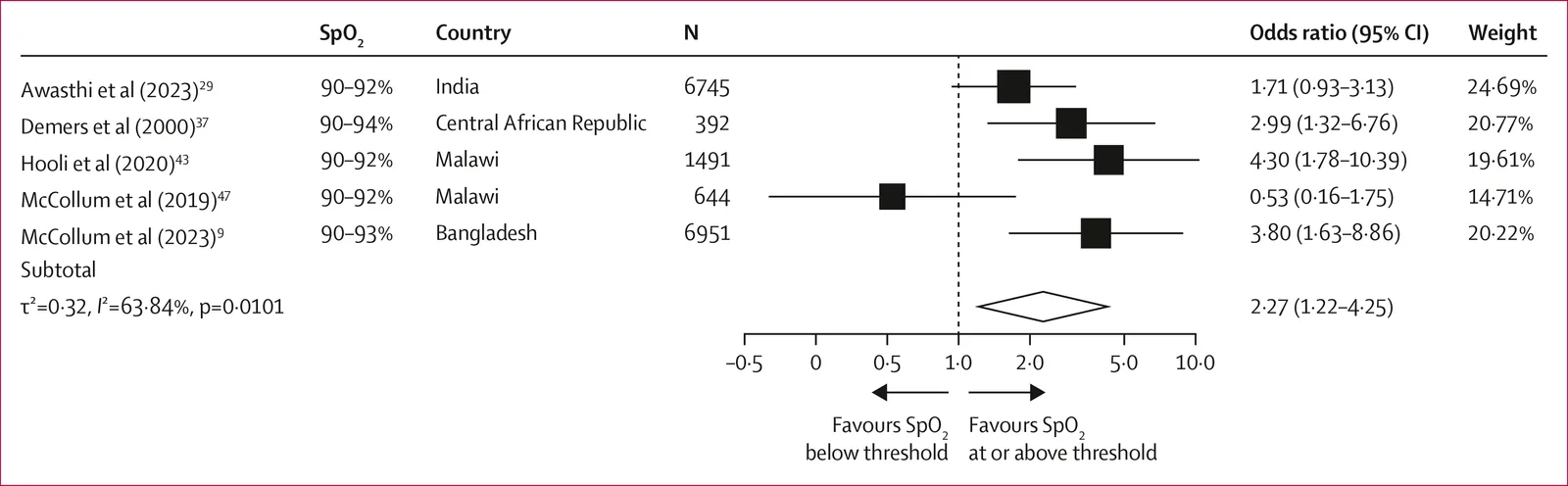
Odds of mortality among children with lower respiratory infection comparing SpO_2_ 90–94% with SpO_2_ 95–100% Based on harmonised study SpO_2_ thresholds. Analyses used the random-effects restricted maximum likelihood model. SpO_2=_peripheral capillary oxyhaemoglobin saturation.

Across studies included in the primary meta-analysis, 48·1% (95% CI 44·8–50·8) of deaths among children with LRI were attributable to hypoxaemia of any SpO_2_ threshold (appendix 1 p 29). The PAF for moderate hypoxaemia (SpO_2_ 90–94%) was 12·2% (95% CI 3·9–16·7).

When studies were stratified by Hib and PCV country vaccination, the association between hypoxaemia and mortality in children with LRI was lowest in countries where both Hib and PCV vaccination had been fully implemented by the start of the study; however, differences between subgroups were not statistically significant (appendix 1 p 24).

For altitude, we included only studies reporting the WHO hypoxaemia threshold (SpO_2_ <90%). Studies conducted at 0–1499 metres (OR 4·88 [95% CI 3·82–6·25]) and 1500–2499 metres (5·32 [2·09–13·53]) above sea level showed strong associations between hypoxaemia and mortality. The single study conducted at ≥2500 metres above sea level showed a smaller association, although this estimate was not statistically significant (OR 1·51 [95% CI 0·75–3·04]). Full results are shown in appendix 1 (p 25).

Similarly, studies using the WHO definition of LRI, enriched WHO definition, and other definitions all showed increased odds of mortality among children with hypoxaemia compared with those without hypoxaemia (appendix 1 p 26).

The sensitivity analysis restricting to studies with low or moderate risk of bias did not materially change the association between hypoxaemia and mortality (appendix 1 p 27). When restricting the analysis to unadjusted ORs, without substituting adjusted ORs or RRs, the pooled estimate was similar to the primary analysis (OR 4·50 [95% CI 3·51–5·76] *vs* 4·36 [3·52–5·39]; appendix 1 p 28).

The risk of bias for the 44 studies included in the systematic review is reported in appendix 1 (pp 30–31). Of the 33 studies included in the primary meta-analysis, eight (24%) had a high overall risk of bias, primarily due to absence of adjustment for potential confounders and concerns about statistical analysis.

Using an adapted GRADE approach, the certainty of evidence was rated as moderate for the overall association between hypoxaemia and mortality and rated as high for the association between moderate hypoxaemia (SpO_2_ 90–94%) and mortality (appendix 1 p 32).

## Discussion

Our systematic review and meta-analysis evaluated 33 studies with 155 633 children to update the estimates of the association between hypoxaemia and mortality among children with LRIs in LMICs. Consistent with the original 2015 meta-analysis, which analysed 12 studies including 13 936 children (OR 5·47 [95% CI 3·93–7·63]), we found that WHO-defined hypoxaemia (SpO_2_ <90%) remains strongly associated with mortality (4·75 [3·42–6·58]). Importantly, we also identified an increase in mortality risk among 16 223 children with SpO_2_ 90–94%, compared with SpO_2_ 95–100% (2·27 [1·22–4·25]), a range currently above the WHO treatment threshold for hypoxaemia. These findings are consistent with a growing body of literature indicating that current childhood pneumonia management guidelines fail to capture an additional group of children at elevated risk of death.^9^^,^^62^, ^63^, ^64^ Although whether this risk can be mitigated by supplemental oxygen or other interventions is unknown, these results underscore the need to re-examine thresholds for clinical management.

Our findings align with a 2025 systematic review and meta-analysis on hypoxaemia prevalence in patients in LMICs, which reported as a secondary outcome that hypoxaemia was associated with higher odds of death (OR 4·56 [95% CI 2·97–7·00]) among 33 285 children aged 1 month to 17 years admitted with primary respiratory conditions across 23 included studies.^65^ This 2025 systematic review did not evaluate different SpO_2_ thresholds, and most included studies used the WHO threshold of <90%. Pooled analysis of outpatient paediatric data has shown elevated mortality risk at SpO_2_ 94–95% compared with 98–100% (1·52 [1·18–1·98]).^62^ We were unable to assess mortality risk in this range due to a small amount of data. Nevertheless, our findings, together with these studies, support a graded, dose-dependent relationship between hypoxaemia severity and mortality.^62^ Evidence is insufficient to assess the precise SpO_2_ measurement at which no significant mortality risk exists.

Per the WHO IMCI algorithm, severe pneumonia requires hospital referral for parenteral antibiotics and is defined as cough or difficulty breathing, or both, accompanied by a general danger sign (eg, inability to breastfeed or drink, vomiting everything, lethargy or reduced unconsciousness, or convulsions).^6^ Importantly, our meta-analysis found that the magnitude of mortality risk for children with SpO_2_ 90–94% compared with SpO_2_ 95–100% (OR 2·27 [95% CI 1·22–4·25]), is similar to the mortality risk associated with a general danger sign (3·1 [1·2–7·9]).^9^^,^^66^ This finding suggests that children with SpO_2_ in the range of 90–94% might warrant similar consideration for referral and management as those with general danger signs, given their elevated risk of death.

Because pulse oximeters non-invasively measure SpO_2_, our findings highlight their crucial role not only in identifying children who need oxygen therapy but also in risk stratification and prognostication. Pulse oximetry can help clinicians recognise children at elevated risk of death—even at SpO_2_ measurements higher than the current WHO treatment threshold—and could therefore inform decisions about who could benefit from closer monitoring or oxygen treatment.

Management of children with an SpO_2_ measurement higher than the WHO threshold (ie, moderate hypoxaemia) warrants careful consideration. Evidence is scarce in terms of whether providing supplemental oxygen, antibiotics, or other therapies to children with SpO_2_ 90–94% improves outcomes. Studies from high-income countries suggest that more conservative SpO_2_ thresholds—lower than WHO-recommended thresholds (eg, SpO_2_ ≥88%)—are safe and do not worsen outcomes, at least for otherwise healthy infants with viral bronchiolitis;^67^, ^68^, ^69^ however, these findings are unlikely to generalise to LMICs, where children face substantially higher baseline risks due to comorbidities such as malnutrition, anaemia, HIV, and tuberculosis, as well as barriers to timely care, delayed presentation, and reduced access to advanced monitoring and critical care services if illness severity worsens.

The Children’s Oxygen Administrative Strategies Trial (COAST) offers additional evidence relevant to oxygen-initiation thresholds.^70^ Conducted in Uganda and Kenya, COAST evaluated several oxygen initiation strategies at varying SpO_2_ thresholds—including low-flow oxygen, high-flow oxygen, and no oxygen treatment (referred to as permissive hypoxaemia)—among hospitalised children aged 28 days to 12 years. The randomised trial was stopped early for futility after enrolling 1852 (45%) of 4200 planned participants. Among children with SpO_2_ 80–91%, the authors reported an OR of 1·16 (95% CI 0·49–2·74; p=0·728) for 48-h mortality (primary outcome) comparing the oxygen (low-flow or high-flow) and no-oxygen groups; however, children with SpO_2_ 90–91% who received low-flow or high-flow oxygen had 85% lower odds of treatment failure at 48 h (secondary outcome, defined as a SpO_2_ <92% plus respiratory distress), compared with those not receiving oxygen (OR 0·15 [95% CI 0·02–0·67]; p=0·0071). The COAST investigators cautioned that their findings were vulnerable to type 2 error due to early termination and thus non-definitive, but suggested that the results raised uncertainty around optimal oxygen-initiation thresholds, including whether permissive hypoxaemia (delaying oxygen initiation) might be a viable strategy for children with SpO_2_ <90%.

When considered alongside our meta-analysis, alternative interpretations of the COAST data emerge. Specifically, the statistically significant reduction in treatment failure at SpO_2_ 90–91%, although a secondary outcome, supports earlier oxygen initiation in children with SpO_2_ ≥90%, rather than delayed treatment. This interpretation aligns with our findings that children with SpO_2_ 90–94% (n=16 223) have elevated mortality risk compared with children with higher saturations. Furthermore, because all COAST participants received hospital-level monitoring, parenteral antibiotics, and supportive care regardless of oxygen therapy, the trial could also be interpreted as supporting hospital-based management, irrespective of oxygen therapy, as a potential strategy for children with SpO_2_ 90–94%. More research is needed to address these key clinical management questions.

These considerations underscore the urgent need for randomised trials in LMICs to assess whether supplemental oxygen or other interventions for children with SpO_2_ ≥90% improve survival and other functional outcomes.^71^ In the meantime, at a minimum, we recommend closer monitoring or follow-up to identify disease progression and prevent adverse outcomes. While more evidence is awaited, available evidence does not preclude careful oxygen therapy for children with SpO_2_ 90–94%. We acknowledge that both closer monitoring and oxygen provision have resource and operational implications, and their implementation will need to be weighed carefully against local capacity. Notably, the use of pulse oximetry remains inconsistent across many LMIC settings, posing a key challenge to identifying hypoxaemia and implementing any SpO_2_ threshold-based management strategy. The strong association between hypoxaemia and mortality shown by our meta-analysis additionally highlights the crucial need for sustained investment in pulse oximetry and oxygen delivery systems. Importantly, the COVID-19 pandemic spurred investment in pulse oximetry, medical oxygen supply, and related infrastructure in LMICs to improve availability and access.^72^^,^^73^ Leveraging these pandemic-driven investments as part of strengthened, long-term oxygen systems could support routine pulse oximetry use, enable earlier detection of hypoxaemia in children, and help prevent avoidable deaths.^74^

This study has several limitations. First, the use of pulse oximetry to measure SpO_2_ in young children and infants is more complex than in older children and adults and requires skill, including proper probe placement, distraction techniques, and careful interpretation of the plethysmograph waveform to assess measurement quality. Most studies did not report who performed measurements, which devices were used, or whether quality controls were implemented. Device performance also varies, with evidence suggesting uncertainties in performance across different skin tones.^75^, ^76^, ^77^, ^78^, ^79^ These factors, along with substantial improvements in pulse oximeter technology over the past decades, should be kept in mind when interpreting these data. Second, included studies reported SpO_2_ at a single time point, limiting our ability to assess dynamic changes over the course of illness. We also could not systematically account for comorbidities such as shock, sepsis, malaria, or heart failure, which can contribute to hypoxaemia and independently alter mortality risk, nor for conditions such as anaemia that can increase mortality risk without affecting SpO_2_. These factors might influence the observed association between SpO_2_ and death. In addition, the original 2015 review excluded studies with fewer than five deaths. Although we did not identify any such studies in our updated search, some older studies could have been excluded for this reason. Although excluding studies with fewer than five deaths could change the magnitude of the SpO_2_–mortality association, the consistency of our findings with similar analyses suggests that the potential for meaningful bias is low. Finally, most included studies were hospital-based. In outpatient or community settings, weak referral systems and delays in care could increase the risk of death among children with hypoxaemia; however, children in these settings could also present earlier with less severe illness and fewer comorbidities, lowering their baseline risk of mortality. Either scenario could alter the observed associations.

In summary, this updated systematic review and meta-analysis confirms that hypoxaemia remains a strong predictor of mortality in children with LRIs in LMICs, despite major improvements in child health over the past decade, including widespread PCV and Hib vaccination roll-out and declines in overall child mortality. Although the highest mortality risk was observed in children with SpO_2_ <90%, a substantially increased risk was also seen in children with SpO_2_ 90–94%. These findings highlight that, even as overall LRI outcomes improve, hypoxaemia continues to signal high mortality risk. Our findings underscore the urgent need to consider alternative hypoxaemia definitions that incorporate higher SpO_2_ thresholds into LRI and pneumonia management guidelines for LMICs.

## Equitable Partnership Declaration

## Data sharing

The study protocol is publicly available at PROSPERO (CRD42023433946). All search strategies and study-level characteristics are reported in the manuscript or supplemental files. Additional extracted data and analytic files are available from the corresponding author upon reasonable request.

## Declaration of interests

HBS reports grant funding to her institution from the National Institutes of Health (NIH) and the US Centers for Disease Control and Prevention. TC reports grants to his institution (UCL) from the NIH and the Wellcome Trust; consulting fees paid to his institution from the Global Fund to Fight AIDS, Tuberculosis and Malaria; support for travel from FWO Belgium; and participation as chair of a trial steering committee for an adolescent mental health study in Nepal. ML reports grant funding to her institutions from the Chiesi Foundation and UNICEF, and participation on WHO and UNICEF advisory boards (pro bono). SH reports grant funding from the National Heart, Lung, and Blood Institute, including a K23 award, and from the Thrasher Research Fund, and serves as an uncompensated member of the Board of Trustees for Harris Health (USA), unrelated to the present work. EDM reports grants from the NIH, the Bill & Melinda Gates Foundation, USAID, the US Centers for Disease Control and Prevention, the Thrasher Research Fund, and Moderna; support for travel to attend a WHO Guideline Development Group meeting in Geneva in 2024; participation on the data safety monitoring board for the Tools for Integrated Management of Child Illness Trial; and a leadership role as co-chair of The Union Child Pneumonia Working Group. All other authors declare no competing interests.
