# Supplementary material for: Hypoxaemia and mortality in children with lower respiratory infection in low-income and middle-income countries: systematic review and meta-analysis
Source: Lancet Glob Health. 2026 Sep 1;14(10):None. doi: 10.1016/j.langlo.2026.104031 (PMC13652795; doi:10.1016/j.langlo.2026.104031)
Supplement: Supplementary file 1 [file mmc1.pdf]

THE LANCET
Global Health

Supplemental appendix 1

This appendix formed part of the original submission and has been peer reviewed. We post it as supplied by the authors.

Supplement to: Hoekstra NE, Joseph KS, Schuh HB, et al. Hypoxaemia and mortality in children with lower respiratory infection in low-income and middle-income countries: systematic review and meta-analysis. *Lancet Glob Health* 2026. https://doi.org/10.1016/j.langlo.2026.104031

Supplementary Material – Table of Contents

Supplement	Page
Table S1: Search Strategy	2-17
File S1: Quality In Prognosis Studies (QUIPS) tool	18-20
File S2: Assessment of the Overall Bias for Each Single Study	21
Table S2: Additional Characteristics of Included Studies	22-23
Figure S1: Odds of mortality from lower respiratory infection among children comparing hypoxemic versus non-hypoxemic groups, stratified by country Hib and PCV vaccine era.	24
Figure S2: Odds of death from lower respiratory infection by study altitude	25
Figure S3: Odds of mortality from lower respiratory infection among children comparing hypoxemic versus non-hypoxemic groups, stratified by study definition of lower respiratory infection	26
Figure S4: Odds of mortality from lower respiratory infection among children comparing hypoxemic versus non-hypoxemic groups, stratified by study risk of bias (low vs. moderate) and overall.	27
Figure S5: Odds of mortality from lower respiratory infection among children comparing oxygen saturation below versus at or above specified SpO_2 thresholds, using unadjusted odds ratios only.	28
Table S3: Population attributable fraction (PAF) for mortality associated with hypoxemia across oxygen saturations	29
Table S4: Risk of Bias in the Included Studies	30-31
Table S5: Summary of Findings: Association Between Hypoxemia and Mortality	32

Table S1. Search Strategy
PubMed

Pneumonia	"Pneumonia"[Mesh] OR pneumonia[tw] OR "respiratory infection"[all fields] OR "respiratory infections"[all fields] OR "lower respiratory tract infections" [all fields] OR bronchiolitis[mesh] OR bronchiolitis, viral[mesh] OR bronchiolitis[all fields]
Children	child[mesh] OR child[tw] OR children[tw] OR pediatrics[mesh] OR paediatrics[all fields] OR paediatric[all fields] OR infant[mesh] OR infant[tw] OR infants[tw]OR newborn[tw] OR newborns[tw] OR neonate[tw] OR neonates[tw]
Risk factors	risk factors[mesh] OR "risk factor"[all fields] OR "risk factors"[all fields] OR determinant[all fields] OR determinants[all fields] OR predictor[all fields] OR predictors[all fields] OR "predictive value"[all fields] OR risk[mesh] OR risk[all fields] OR risks[all fields]
Hypoxemia	hypoxemia[all fields] OR hypoxaemia[all fields] OR hypoxia[mesh] OR hypoxia[all fields] OR "oxygen saturation"[mesh] OR "oxygen saturation"[all fields]
Mortality	mortality[mesh] OR mortality[subheading] OR mortality[tw] OR death[mesh] OR deaths[all fields] OR fatality[all fields] OR fatal[all fields]
LMIC	("Afghanistan"[mh] OR "Afghanistan"[tw] OR "Afghan"[tw] OR "Afghani"[tw] OR "Afghans"[tw] OR "Africa"[mh] OR "Africa"[tw] OR "African"[tw] OR "Africans"[tw] OR "Africana"[tw] OR "Albania"[mh] OR "Albania"[tw] OR "Albanian"[tw] OR "Albanians"[tw] OR "Algeria"[mh] OR "Algeria"[tw] OR "Algerian"[tw] OR "Algerians"[tw] OR "Angola"[mh] OR "Angola"[tw] OR "Angolan"[tw] OR "Angolans"[tw] OR "Antigua and Barbuda"[mh] OR "Antigua"[tw] OR "Barbuda"[tw] OR "Antiguan"[tw] OR "Antiguans"[tw] OR "Barbudan"[tw] OR "Argentina"[mh] OR "Argentina"[tw] OR "Argentinian"[tw] OR "Argentinians"[tw] OR "Argentinos"[tw] OR "Argentine"[tw] OR "Armenia"[mh] OR "Armenia"[tw] OR "Armenian"[tw] OR "Armenians"[tw] OR "Aruba"[mh] OR "aruba"[tw] OR "Aruban"[tw] OR "Arubans"[tw] OR "Azerbaijan"[mh] OR "Azerbaijan"[tw] OR "Azerbaijani"[tw] OR "Azerbaijanis"[tw] OR "Azerbaijanian"[tw] OR "Azerbaijanians"[tw] OR "Bahrain"[mh] OR "Bahrain"[tw] OR "Bahrainian"[tw] OR "Bahrainians"[tw] OR "Bahraini"[tw] OR "Bangladesh"[mh] OR "Bangladesh"[tw] OR "Bangladeshi"[tw] OR "Bengalese"[tw] OR "Barbados"[mh] OR "Barbados"[tw] OR "Barbadian"[tw] OR "Barbadians"[tw] OR "Bajan"[tw] OR "Republic of Belarus"[mh] OR "Belarus"[tw] OR "Belarusian"[tw] OR "Belarusians"[tw] OR "Belarussian"[tw] OR "Belorus"[tw] OR "Belorussia"[tw] OR "Belorussian"[tw] OR "Belorussians"[tw] OR "Byelarus"[tw] OR "Byelorus"[tw] OR "Byelorussia"[tw] OR "Byelorussian"[tw] OR "Byelorussians"[tw] OR "Belize"[mh] OR "Belize"[tw] OR "Belizean"[tw] OR "British Honduras"[tw] OR "Benin"[mh] OR "benin"[tw] OR "Dahomey"[tw] OR "Bhutan"[mh] OR "Bhutan"[tw] OR "Bolivia"[mh] OR "Bolivia"[tw] OR "Bolivian"[tw] OR "Bolivians"[tw] OR "Bosnia and Herzegovina"[mh] OR "Bosnia"[tw] OR "Bosnian"[tw] OR "Bosnians"[tw] OR

	"Herzegovina"[tw] OR "Hercegovina"[tw] OR "Botswana"[mh] OR "Botswana"[tw] OR "Botswanan"[tw] OR "Batswana"[tw] OR "Bechuanaland"[tw] OR "Brazil"[mh] OR "Brazil"[tw] OR "Brasil"[tw] OR "Brasilia"[tw] OR "Brazilian"[tw] OR "Brazilians"[tw] OR "Brasilian"[tw] OR "Brasilians"[tw] OR "Bulgaria"[mh] OR "Bulgaria"[tw] OR "Bulgarian"[tw] OR "Bulgarians"[tw] OR "Burkina Faso"[mh] OR "Burkina"[tw] OR "burkinese"[tw] OR "upper volta"[tw] OR "Burkinabe"[tw] OR "Burundi"[mh] OR "Burundi"[tw] OR "Burundian"[tw] OR "Urundi"[tw] OR "Cabo Verde"[mh] OR "cabo verde"[tw] OR "cape verde"[tw] OR "Cabo Verde people"[nm] OR "Cape Verdean"[tw] OR "Cabo Verdean"[tw] OR "Cambodia"[mh] OR "Cambodia"[tw] OR "Cambodian"[tw] OR "Cambodians"[tw] OR "Kampuchea"[tw] OR "Kampuchean"[tw] OR "Kampucheans"[tw] OR "khmer"[tw] OR "khmers"[tw] OR "Cameroon"[mh] OR "Cameroon"[tw] OR "Cameroons"[tw] OR "Cameroun"[tw] OR "Camerouns"[tw] OR "Cameroonian"[tw] OR "Cameroonians"[tw] OR "Camerounian"[tw] OR "Camerounians"[tw] OR "Cameroonese"[tw] OR "Central African Republic"[mh] OR "Central African Republic"[tw] OR "ubangi shari"[tw] OR "Chad"[mh] OR "Chad"[tw] OR "Chile"[mh] OR "Chile"[tw] OR "Chilean"[tw] OR "Chileans"[tw] OR "China"[mh] OR "China"[tw] OR "Chinese"[tw] OR "Manchuria"[tw] OR "Colombia"[mh] OR "Colombia"[tw] OR "Colombian"[tw] OR "Colombians"[tw] OR "Comoros"[mh] OR "Comoro"[tw] OR "Comoros"[tw] OR "Comore"[tw] OR "comores"[tw] OR "Comorian"[tw] OR "Comorians"[tw] OR "mayotte"[tw] OR "Congo"[mh] OR "Congo"[tw] OR "Congolese"[tw] OR "Democratic Republic of the Congo"[mh] OR "zaire"[tw] OR "Zaireen"[tw] OR "Zairean"[tw] OR "Katanga"[tw] OR "Costa Rica"[mh] OR "Costa Rica"[tw] OR "Costa Rican"[tw] OR "Cote d'Ivoire"[mh] OR "Cote d'Ivoire"[tw] OR "Cote d' Ivoire"[tw] OR "Cote Divoire"[tw] OR "Cote d Ivoire"[tw] OR "Cote dIvoire"[tw] OR "Ivory Coast"[tw] OR "Ivorian"[tw] OR "Croatia"[mh] OR "Croatia"[tw] OR "Croatian"[tw] OR "Croatians"[tw] OR "Yugoslavia"[tw] OR "Yugoslavian"[tw] OR "Cuba"[mh] OR "cuba"[tw] OR "Cuban"[tw] OR "Cubans"[tw] OR "Cuba's"[tw] OR "Cyprus"[mh] OR "Cyprus"[tw] OR "Cypriot"[tw] OR "Cypriots"[tw] OR "Cypriote"[tw] OR "Czech Republic"[mh] OR "Czech"[tw] OR "Czechs"[tw] OR "Czechoslovakia"[tw] OR "Czechoslovakian"[tw] OR "Czechoslovakians"[tw] OR "Czechia"[tw] OR "Czechoslowacka"[tw] OR "Djibouti"[mh] OR "Djibouti"[tw] OR "Djiboutian"[tw] OR "French Somaliland"[tw] OR "Dominica"[mh] OR "Dominica"[tw] OR "Dominican"[tw] OR "Dominicans"[tw] OR "Ecuador"[mh] OR "Ecuador"[tw] OR "Ecuadorean"[tw] OR "Ecuadoreans"[tw] OR "Ecuadorian"[tw] OR "Ecuadorians"[tw] OR "Ecuadoran"[tw] OR "Ecuadorans"[tw] OR "Egypt"[mh] OR "Egypt"[tw] OR "Egyptian"[tw] OR "Egyptians"[tw] OR "United Arab Republic"[tw] OR "El Salvador"[mh] OR "El Salvador"[tw] OR "Salvadoran"[tw] OR "Salvadorans"[tw] OR "Guinea"[mh] OR "Guinea"[tw] OR "Guinean"[tw] OR "Guineans"[tw] OR "Equatorial Guinea"[mh] OR "Equatoguinea"[tw] OR "Equatoguinean"[tw] OR "Equatoguineans"[tw] OR "Guinea-Bissau"[mh] OR "Guinea Bissau"[tw] OR "GuineaBissau"[tw] OR "Eritrea"[mh] OR "Eritrea"[tw] OR "Eritrean"[tw] OR "Eritreans"[tw] OR "Estonia"[mh]

	OR "Estonia"[tw] OR "Estonian"[tw] OR "Estonians"[tw] OR "Eswatini"[mh] OR "Eswatini"[tw] OR "Eswatinian"[tw] OR "Swaziland"[tw] OR "Swazi"[tw] OR "Swati"[tw] OR "Ethiopia"[mh] OR "Ethiopia"[tw] OR "Ethiopian"[tw] OR "Ethiopians"[tw] OR "Fiji"[mh] OR "Fiji"[tw] OR "Fijan"[tw] OR "Fijian"[tw] OR "Fijians"[tw] OR "Gabon"[mh] OR "Gabon"[tw] OR "Gabonese"[tw] OR "Gabonaise"[tw] OR "Gambia"[mh] OR "Gambia"[tw] OR "Gambian"[tw] OR "Gambians"[tw] OR "Georgia (Republic)"[mh] OR (("Georgia"[tw] OR "Georgian"[tw] OR "Georgians"[tw]) NOT (atlanta[tw] OR California[tw] OR florida[tw])) OR "Ghana"[mh] OR "Ghana"[tw] OR "Ghanian"[tw] OR "Ghanians"[tw] OR "Ghanaian"[tw] OR "Gibraltar"[mh] OR "Gibraltar"[tw] OR "Greece"[mh] OR "Greece"[tw] OR "Greek"[tw] OR "Greeks"[tw] OR "Grecian"[tw] OR "Grenada"[mh] OR "Grenada"[tw] OR "Grenadas"[tw] OR "Grenadian"[tw] OR "Grenadians"[tw] OR "Guam"[mh] OR "Guam"[tw] OR "Guatemala"[mh] OR "Guatemala"[tw] OR "Guatemalan"[tw] OR "Guatemalans"[tw] OR "Guyana"[mh] OR "Guyana"[tw] OR "Guyanas"[tw] OR "Guyanan"[tw] OR "Guiana"[tw] OR "Guianas"[tw] OR "Guianan"[tw] OR "Guyanese"[tw] OR "Haiti"[mh] OR "Haiti"[tw] OR "Haitian"[tw] OR "Haitians"[tw] OR "Hispaniola"[tw] OR "Honduras"[mh] OR "Honduras"[tw] OR "Honduran"[tw] OR "Hondurans"[tw] OR "Hungary"[mh] OR "Hungary"[tw] OR "Hungarian"[tw] OR "Hungarians"[tw] OR "India"[mh] OR "India"[tw] OR "Indian"[tw] OR "Indians"[tw] OR "Indonesia"[mh] OR "Indonesia"[tw] OR "Indonesias"[tw] OR "Indonesian"[tw] OR "Indonesians"[tw] OR "Bali"[tw] OR "Sumatra"[tw] OR "Sumatran"[tw] OR "Malay archipelago"[tw] OR "Timor"[tw] OR "Iran"[mh] OR "Iran"[tw] OR "Iranian"[tw] OR "Iranians"[tw] OR "Iraq"[mh] OR "Iraq"[tw] OR "Iraqi"[tw] OR "Iraqis"[tw] OR "Isle of man"[tw] OR "Jamaica"[mh] OR "Jamaica"[tw] OR "Jamaican"[tw] OR "Jamaicans"[tw] OR "Jordan"[mh] OR "Jordan"[tw] OR "Jordanian"[tw] OR "Jordanians"[tw] OR "Kazakhstan"[mh] OR "Kazakhstan"[tw] OR "Kazakh"[tw] OR "Kazakhs"[tw] OR "Kazakstani"[tw] OR "Kenya"[mh] OR "Kenya"[tw] OR "Kenyan"[tw] OR "Kenyans"[tw] OR "Karabati"[tw] OR "Gilbert Island"[tw] OR "Gilbert Islands"[tw] OR "Democratic People's Republic of Korea"[mh] OR "Korea"[tw] OR "Korean"[tw] OR "Koreans"[tw] OR "Kosovo"[mh] OR "Kosovo"[tw] OR "Kosova"[tw] OR "Kosovar"[tw] OR "Kosovan"[tw] OR "Kyrgyzstan"[mh] OR "Kyrgyz"[tw] OR "Kyrgyzstan"[tw] OR "Kyrgyzstani"[tw] OR "Kyrgyzstanis"[tw] OR "Kyrgyzatan"[tw] OR "Kyrgyzystan"[tw] OR "Kyrgyzistan"[tw] OR "Kirgiz"[tw] OR "Kirgizia"[tw] OR "Kirgizs"[tw] OR "Kirgizstan"[tw] OR "Kirgizstani"[tw] OR "Kirgizstanis"[tw] OR "Kirghiz"[tw] OR "Kirghizs"[tw] OR "Kirghizia"[tw] OR "Kirghizstan"[tw] OR "Kirghizstani"[tw] OR "Kirghizstanis"[tw] OR "Laos"[mh] OR "Laos"[tw] OR "Lao"[tw] OR "Laotian"[tw] OR "Laotian"[tw] OR "Laotians"[tw] OR "Latvia"[mh] OR "Latvia"[tw] OR "Latvian"[tw] OR "Latvians"[tw] OR "Lebanon"[mh] OR "Lebanon"[tw] OR "Lebanese"[tw] OR "Lesotho"[mh] OR "Lesotho"[tw] OR "Lesothan"[tw] OR "Lesothonian"[tw] OR "Basutoland"[tw] OR "Mosotho"[tw] OR "Basotho"[tw] OR "Liberia"[mh] OR "Liberia"[tw] OR "Liberian"[tw]

	OR "Liberians"[tw] OR "Libya"[mh] OR "Libya"[tw] OR "Jamahiriya"[tw] OR "Libyan"[tw] OR "Libyans"[tw] OR "Lithuania"[mh] OR "Lithuania"[tw] OR "Lithuanian"[tw] OR "Lithuanians"[tw] OR "Republic of North Macedonia"[mh] OR "Macedonia"[tw] OR "Macedonian"[tw] OR "Macedonians"[tw] OR "Madagascar"[mh] OR "Madagascar"[tw] OR "Madagascan"[tw] OR "Madagascans"[tw] OR "Malagasy"[tw] OR "Malawi"[mh] OR "Malawi"[tw] OR "Malawian"[tw] OR "Malawians"[tw] OR "Nyasaland"[tw] OR "Malaysia"[mh] OR "Malaysia"[tw] OR "Malaysian"[tw] OR "Malaysians"[tw] OR "Malay"[tw] OR "Malayali"[tw] OR "Malayalian"[tw] OR "Maldives"[mh] OR "Maldives"[tw] OR "Maldivian"[tw] OR "Maldivians"[tw] OR "Indian Ocean"[mh] OR "Indian ocean"[tw] OR "Indic Ocean"[tw] OR "Mali"[mh] OR "Mali"[tw] OR "Malian"[tw] OR "Malians"[tw] OR "mali's"[tw] OR "Malta"[mh] OR "Malta"[tw] OR "Maltese"[tw] OR "Malta's"[tw] OR "Micronesia"[mh] OR "Micronesia"[tw] OR "Micronesian"[tw] OR "Micronesians"[tw] OR "Marshallese"[tw] OR "Kiribati"[tw] OR "Marshall island"[tw] OR "Marshall Islander"[tw] OR "Marshall Islanders"[tw] OR "Marshall Islands"[tw] OR "Nauru"[tw] OR "Nauran"[tw] OR "Nauruans"[tw] OR "Naurian's"[tw] OR "Mariana"[tw] OR "Marianas"[tw] OR "Palau"[mh] OR "Palau"[tw] OR "Paluan"[tw] OR "Palauns"[tw] OR "Tuvalu"[tw] OR "Mauritania"[mh] OR "Mauritania"[tw] OR "Mauritanian"[tw] OR "Mauritanians"[tw] OR "Mauritius"[tw] OR "Mexico"[mh] OR "Mexico"[tw] OR "Mexican"[tw] OR "Mexicans"[tw] OR "Moldova"[mh] OR "Moldova"[tw] OR "Moldovia"[tw] OR "Moldovan"[tw] OR "Moldovans"[tw] OR "Moldovian"[tw] OR "Moldovians"[tw] OR "Mongolia"[mh] OR "Mongolia"[tw] OR "Mongol"[tw] OR "Mongols"[tw] OR "Mongolian"[tw] OR "Mongolians"[tw] OR "Mongoloid"[tw] OR "Mongoloids"[tw] OR "Mongolic"[tw] OR "Montenegro"[mh] OR "Montenegro"[tw] OR "Morocco"[mh] OR "Morocco"[tw] OR "Moroccan"[tw] OR "Moroccans"[tw] OR "Ifni"[tw] OR "Mozambique"[mh] OR "Mozambique"[tw] OR "Mozambican"[tw] OR "Mozambicans"[tw] OR "Myanmar"[mh] OR "Myanmar"[tw] OR "Burma"[tw] OR "Burmese"[tw] OR "Namibia"[mh] OR "Namibia"[tw] OR "Namibian"[tw] OR "Namibians"[tw] OR "Nepal"[mh] OR "Nepal"[tw] OR "Nepalese"[tw] OR "Nepali"[tw] OR "New Caledonia"[mh] OR "New Caledonia"[tw] OR "New Caledonian"[tw] OR "New Caledonians"[tw] OR "Netherlands Antilles"[mh] OR "Netherlands Antilles"[tw] OR "Antillean"[tw] OR "Antilleans"[tw] OR "Antillian"[tw] OR "Antillians"[tw] OR "Dutch Antilles"[tw] OR "Nicaragua"[mh] OR "Nicaragua"[tw] OR "Nicaraguan"[tw] OR "Nicaraguans"[tw] OR "Niger"[mh] OR "Niger"[tw] OR "Nigerien"[tw] OR "Nigeriens"[tw] OR "Oman"[mh] OR "Oman"[tw] OR "Omani"[tw] OR "Omanis"[tw] OR "oman's"[tw] OR "Pakistan"[mh] OR "Pakistan"[tw] OR "Pakistani"[tw] OR "Pakistanis"[tw] OR "Pakistanian"[tw] OR "Pakistanese"[tw] OR "Palestine"[tw] OR "Palestinian"[tw] OR "Palestinians"[tw] OR "Gaza"[tw] OR "Gazan"[tw] OR "Gazans"[tw] OR "West Bank"[tw] OR "Panama"[mh] OR "Panama"[tw] OR "Panamanian"[tw] OR "Panamanians"[tw] OR "Paraguay"[mh] OR "Paraguay"[tw] OR "Paraguayan"[tw] OR "Paraguayans"[tw] OR "Paraguayo"[tw] OR

	"Peru"[mh] OR "Peru"[tw] OR "Peruvian"[tw] OR "Peruvians"[tw] OR "peru's"[tw] OR "Philippines"[mh] OR "Philippine"[tw] OR "Philippines"[tw] OR "Philipine"[tw] OR "Philipines"[tw] OR "Phillipine"[tw] OR "Phillipines"[tw] OR "Phillippine"[tw] OR "Phillippines"[tw] OR "Filipino"[tw] OR "Filipinos"[tw] OR "Filipina"[tw] OR "Filipinas"[tw] OR "Poland"[mh] OR "Poland"[tw] OR "Polish"[tw] OR "Pole"[tw] OR "Poles"[tw] OR "Portugal"[mh] OR "Portugal"[tw] OR "Portuguese"[tw] OR "Puerto Rico"[mh] OR "Puerto Rico"[tw] OR "Puerto Rican"[tw] OR "Puerto Ricans"[tw] OR "Romania"[mh] OR "Romania"[tw] OR "Romanian"[tw] OR "Romanians"[tw] OR "Russia"[mh] OR "Russia"[tw] OR "USSR"[tw] OR "Soviet"[tw] OR "Soviets"[tw] OR "Russian"[tw] OR "Russians"[tw] OR "Rwanda"[mh] OR "Rwanda"[tw] OR "Rwandan"[tw] OR "Rwandans"[tw] OR "Rwandese"[tw] OR "Ruanda"[tw] OR "Ruandan"[tw] OR "Ruandans"[tw] OR "Ruandese"[tw] OR "Samoa"[mh] OR "Samoa"[tw] OR "Samoan"[tw] OR "Samoans"[tw] OR "Navigator Island"[tw] OR "Navigator Islander"[tw] OR "Navigator Islanders"[tw] OR "Pacific island"[tw] OR "Pacific Islands"[tw] OR "Pacific Islander"[tw] OR "Pacific Islanders"[tw] OR "Polynesia"[mh] OR "Polynesia"[tw] OR "Polynesian"[tw] OR "Polynesians"[tw] OR "Sao Tome and Principe"[mh] OR "Sao Tome and Principe"[tw] OR "Sao Tome e Principe"[tw] OR "Sao Tomean"[tw] OR "Sao Tomeans"[tw] OR "Santomean"[tw] OR "Santomeans"[tw] OR "Saudi Arabia"[mh] OR "Saudi Arabia"[tw] OR "Saudi Arabian"[tw] OR "Saudi Arabians"[tw] OR "Saudi"[tw] OR "Saudis"[tw] OR "Senegal"[mh] OR "Senegal"[tw] OR "Senegalese"[tw] OR "Serbia"[mh] OR "Serbia"[tw] OR "Serbian"[tw] OR "Serbians"[tw] OR "Seychelles"[mh] OR "Seychelles"[tw] OR "Seychelle Islands"[tw] OR "Seychellois"[tw] OR "Sierra Leone"[mh] OR "Sierra Leone"[tw] OR "Sierra Leonean"[tw] OR "Slovakia"[mh] OR "Slovakia"[tw] OR "Slovakian"[tw] OR "Slovakians"[tw] OR "Slovak"[tw] OR "Slovaks"[tw] OR "Slovenia"[mh] OR "Slovenia"[tw] OR "Slovenian"[tw] OR "Slovenians"[tw] OR "Melanesia"[mh] OR "Melanesia"[tw] OR "Melanesian"[tw] OR "Melanesians"[tw] OR "Solomon island"[tw] OR "Solomon Islands"[tw] OR "Solomon Islander"[tw] OR "Solomon Islanders"[tw] OR "Norfolk Island"[tw] OR "Norfolk Islands"[tw] OR "Norfolk Islander"[tw] OR "Norfolk Islanders"[tw] OR "Somalia"[mh] OR "Somalia"[tw] OR "Somali"[tw] OR "Somalis"[tw] OR "Somalian"[tw] OR "Somalians"[tw] OR "Somaliland"[tw] OR "Sri Lanka"[mh] OR "Sri Lanka"[tw] OR "Ceylon"[tw] OR "Ceylonese"[tw] OR "Sri Lankan"[tw] OR "Sri Lankans"[tw] OR "Saint Kitts and Nevis"[mh] OR "Saint Kitts and Nevis"[tw] OR "St Kitts and Nevis"[tw] OR "Kittian"[tw] OR "Nevisian"[tw] OR "Saint Lucia"[mh] OR "Saint Lucia"[tw] OR "St Lucia"[tw] OR "Saint Lucian"[tw] OR "St Lucian"[tw] OR "Saint Vincent and the Grenadines"[mh] OR "Saint Vincent"[tw] OR "St Vincent"[tw] OR "Vincentian"[tw] OR "Vincentians"[tw] OR "Grenadine"[tw] OR "Grenadines"[tw] OR "Grenadinian"[tw] OR "Grenadinians"[tw] OR "Sudan"[mh] OR "Sudan"[tw] OR "Sudanese"[tw] OR "Sudanian"[tw] OR "South Sudan"[mh] OR "Suriname"[mh] OR "Suriname"[tw] OR "Surinam"[tw] OR "Surinamese"[tw] OR "Syria"[mh] OR "Syria"[tw] OR "Syrian"[tw]

	OR "Syrians"[tw] OR "Tajikistan"[mh] OR "Tajikistan"[tw] OR "Tadjikistan"[tw] OR "Tadzhikistan"[tw] OR "Tajik"[tw] OR "Tajiks"[tw] OR "Tanzania"[mh] OR "Tanzania"[tw] OR "Tanganyika"[tw] OR "Zanzibar"[tw] OR "Tanzanian"[tw] OR "Tanzanians"[tw] OR "Tanganyikan"[tw] OR "Thailand"[mh] OR "Thailand"[tw] OR "Thai"[tw] OR "Thais"[tw] OR "Timor-Leste"[mh] OR "Timor Leste"[tw] OR "Timorleste"[tw] OR "East Timor"[tw] OR "Timorese"[tw] OR "Maubere"[tw] OR "Timor Island"[tw] OR "Togo"[mh] OR "Togo"[tw] OR "Togolese"[tw] OR "togo's"[tw] OR "Tonga"[mh] OR "Tonga"[tw] OR "Tongan"[tw] OR "Tongans"[tw] OR "Trinidad and Tobago"[mh] OR "Trinidad"[tw] OR "Tobago"[tw] OR "Trinidadian"[tw] OR "Trinidadians"[tw] OR "Tobagonian"[tw] OR "Tobagonians"[tw] OR "Tunisia"[mh] OR "Tunisia"[tw] OR "Tunisian"[tw] OR "Tunisians"[tw] OR "Turkey"[mh] OR "Turkiye"[tw] OR "Turkey"[tw] OR "Turk"[tw] OR "Turks"[tw] OR "Turkish"[tw] OR "Turkmen"[tw] OR "Turkmens"[tw] OR "Uganda"[mh] OR "Uganda"[tw] OR "Ugandan"[tw] OR "Ugandans"[tw] OR "Ukraine"[mh] OR "Ukraine"[tw] OR "Ukrainian"[tw] OR "Ukrainians"[tw] OR "Uruguay"[mh] OR "Uruguay"[tw] OR "Uruguayan"[tw] OR "Uruguayans"[tw] OR "Uzbekistan"[mh] OR "Uzbekistan"[tw] OR "Uzbekistani"[tw] OR "Uzbekistanis"[tw] OR "Uzbek"[tw] OR "Uzbeks"[tw] OR "Vanuatu"[mh] OR "Vanuatu"[tw] OR "New Hebrides"[tw] OR "Ni-Vanuatu"[tw] OR "Vanuatuan"[tw] OR "Vanuatuans"[tw] OR "Venezuela"[mh] OR "Venezuela"[tw] OR "Venezuelan"[tw] OR "Venezuelans"[tw] OR "Vietnam"[mh] OR "Vietnam"[tw] OR "Viet Nam"[tw] OR "Vietnamese"[tw] OR "Viet namese"[tw] OR "Yemen"[mh] OR "Yemen"[tw] OR "Yemeni"[tw] OR "Yemenite"[tw] OR "Yemenis"[tw] OR "Yugoslavia"[mh] OR "Yugoslavia"[tw] OR "Yugoslavian"[tw] OR "Yugoslav"[tw] OR "Yugoslavs"[tw] OR "Zambia"[mh] OR "Zambia"[tw] OR "Zambian"[tw] OR "Zambians"[tw] OR "Zimbabwe"[mh] OR "Zimbabwe"[tw] OR "Rhodesia"[tw] OR "Zimbabwean"[tw] OR "Zimbabweans"[tw] OR "Rhodesian"[tw] OR "Rhodesians"[tw] OR "Arab country"[tw] OR "Arab countries"[tw] OR "Middle East"[mh:noexp] OR "Middle East"[tw] OR "Middle Eastern"[tw] OR "global south"[tw] OR "Sahara"[tw] OR "Saharan"[tw] OR "Subsahara"[tw] OR "Subsaharan"[tw] OR "Maghreb"[tw] OR "Magreb"[tw] OR "Maghrib"[tw] OR "Magrib"[tw] OR "Maghrebin"[tw] OR "Magrebin"[tw] OR "Mahgribin"[tw] OR "Magribin"[tw] OR "Maghrebian"[tw] OR "Magrebian"[tw] OR "Mahgribian"[tw] OR "Magribian"[tw] OR "Maghrebi"[tw] OR "Maghrebis"[tw] OR "Magrebi"[tw] OR "Magrebis"[tw] OR "West Indies"[mh] OR "West Indies"[tw] OR "Caribbean Region"[mh] OR "Caribbean"[tw] OR "Caribbeans"[tw] OR "Central America"[mh] OR "Central America"[tw] OR "Central American"[tw] OR "Central Americans"[tw] OR "Central America"[tiab:~3] OR "Central American"[tiab:~3] OR "Central Americans"[tiab:~3] OR "Latin America"[mh] OR "Latin America"[tw] OR "Latin American"[tw] OR "Latin Americans"[tw] OR "Latin America"[tiab:~3] OR "Latin American"[tiab:~3] OR "Latin Americans"[tiab:~3] OR "South America"[mh] OR "South America"[tw] OR "South American"[tw] OR "South Americans"[tw] OR "South America"[tiab:~3] OR "South

	American"[tiab:~3] OR "South Americans"[tiab:~3] OR "Asia, Central"[mh] OR "Central Asia"[tw] OR "Central Asian"[tw] OR "Central Asians"[tw] OR "Central Asia"[tiab:~3] OR "Central Asian"[tiab:~3] OR "Central Asians"[tiab:~3] OR "Asia, Northern"[mh] OR "North Asia"[tw] OR "North Asian"[tw] OR "North Asians"[tw] OR "North Asia"[tiab:~3] OR "North Asian"[tiab:~3] OR "North Asians"[tiab:~3] OR "Northern Asia"[tw] OR "Northern Asian"[tw] OR "Northern Asians"[tw] OR "Northern Asia"[tiab:~3] OR "Northern Asian"[tiab:~3] OR "Northern Asians"[tiab:~3] OR "Asia, Southeastern"[mh] OR "Southeastern Asia"[tw] OR "Southeastern Asian"[tw] OR "Southeastern Asians"[tw] OR "Southeastern Asia"[tiab:~3] OR "Southeastern Asian"[tiab:~3] OR "Southeastern Asians"[tiab:~3] OR "South Eastern Asia"[tw] OR "South Eastern Asian"[tw] OR "South Eastern Asians"[tw] OR "South Eastern Asia"[tiab:~3] OR "South Eastern Asian"[tiab:~3] OR "South Eastern Asians"[tiab:~3] OR "Southeast Asia"[tw] OR "Southeast Asian"[tw] OR "Southeast Asians"[tw] OR "Southeast Asia"[tiab:~3] OR "Southeast Asian"[tiab:~3] OR "Southeast Asians"[tiab:~3] OR "South East Asia"[tw] OR "South East Asian"[tw] OR "South East Asians"[tw] OR "South East Asia"[tiab:~3] OR "South East Asian"[tiab:~3] OR "South East Asians"[tiab:~3] OR "Asia, Western"[mh:noexp] OR "West Asia"[tw] OR "West Asian"[tw] OR "West Asians"[tw] OR "West Asia"[tiab:~3] OR "West Asian"[tiab:~3] OR "West Asians"[tiab:~3] OR "Western Asia"[tw] OR "Western Asian"[tw] OR "Western Asians"[tw] OR "Western Asia"[tiab:~3] OR "Western Asian"[tiab:~3] OR "Western Asians"[tiab:~3] OR "Europe, Eastern"[mh] OR "East Europe"[tw] OR "Eastern Europe"[tw] OR "East European"[tw] OR "East Europeans"[tw] OR "Eastern European"[tw] OR "Eastern Europeans"[tw] OR "Developing countries"[mh] OR "developing country"[tw] OR "developing countries"[tw] OR "developing nation"[tw] OR "developing nations"[tw] OR "developing population"[tw] OR "developing populations"[tw] OR "developing world"[tw] OR "less developed country"[tw] OR "less developed countries"[tw] OR "less developed nation"[tw] OR "less developed nations"[tw] OR "less developed world"[tw] OR "lesser developed country"[tw] OR "lesser developed countries"[tw] OR "lesser developed nation"[tw] OR "lesser developed nations"[tw] OR "lesser developed world"[tw] OR "under developed country"[tw] OR "under developed countries"[tw] OR "under developed nation"[tw] OR "under developed nations"[tw] OR "under developed world"[tw] OR "underdeveloped country"[tw] OR "underdeveloped nation"[tw] OR "underdeveloped nations"[tw] OR "underdeveloped world"[tw] OR "middle income country"[tw] OR "middle income country"[tiab:~3] OR "middle income countries"[tw] OR "middle income countries"[tiab:~3] OR "middle income nation"[tw] OR "middle income nations"[tw] OR "middle income population"[tw] OR "middle income populations"[tw] OR "low income country"[tw] OR "low income country"[tiab:~3] OR "low income countries"[tw] OR "low income countries"[tiab:~3] OR "low income nation"[tw] OR "low income nations"[tw] OR "low income population"[tw] OR "low income populations"[tw] OR "lower income country"[tw] OR "lower income country"[tiab:~3] OR "lower income countries"[tw] OR "lower

	income countries"[tiab:~3] OR "lower income nation"[tw] OR "lower income nations"[tw] OR "lower income population"[tw] OR "lower income populations"[tw] OR "underserved country"[tw] OR "underserved countries"[tw] OR "underserved nation"[tw] OR "underserved nations"[tw] OR "underserved population"[tw] OR "underserved populations"[tw] OR "under served population"[tw] OR "under served populations"[tw] OR "under served nation"[tw] OR "under served nations"[tw] OR "under served population"[tw] OR "under served populations"[tw] OR "deprived country"[tw] OR "deprived countries"[tw] OR "deprived population"[tw] OR "deprived populations"[tw] OR "high burden country"[tw] OR "high burden countries"[tw] OR "high burden nation"[tw] OR "high burden nations"[tw] OR "countdown country"[tw] OR "countdown countries"[tw] OR "countdown nation"[tw] OR "countdown nations"[tw] OR "poor country"[tw] OR "poor countries"[tw] OR "poor nation"[tw] OR "poor nations"[tw] OR "poor population"[tw] OR "poor populations"[tw] OR "poor world"[tw] OR "poorer country"[tw] OR "poorer countries"[tw] OR "poorer nation"[tw] OR "poorer nations"[tw] OR "poorer population"[tw] OR "poorer populations"[tw] OR "poorer world" OR "developing economy"[tw] OR "developing economies"[tw] OR "less developed economy"[tw] OR "less developed economies"[tw] OR "underdeveloped economy"[tw] OR "underdeveloped economies"[tw] OR "under developed economy"[tw] OR "under developed economies"[tw] OR "middle income economy"[tw] OR "middle income economies"[tw] OR "low income economy"[tw] OR "low income economies"[tw] OR "lower income economy"[tw] OR "lower income economies"[tw] OR "low gdp"[tw] OR "low gnp"[tw] OR "low gross domestic"[tw] OR "low gross national"[tw] OR "lower gdp"[tw] OR "lower gnp"[tw] OR "lower gross domestic"[tw] OR "lower gross national"[tw] OR "lmic"[tw] OR "lmics"[tw] OR "third world"[tw] OR "lami country"[tw] OR "lami countries"[tw] OR "transitional country"[tw] OR "transitional countries"[tw] OR "emerging economy"[tw] OR "emerging economies"[tw] OR "emerging nation"[tw] OR "emerging nations"[tw])

Embase

Pneumonia	'pneumonia'/exp OR 'bronchiolitis'/exp OR 'viral bronchiolitis'/exp OR pneumonia:ti,ab,kw,de OR 'respiratory infection' OR 'respiratory infections' OR 'lower respiratory tract infections' OR bronchiolitis
Children	'child'/exp OR 'infant'/exp OR 'pediatrics'/exp OR child:ti,ab,kw,de OR children:ti,ab,kw,de OR infant:ti,ab,kw,de OR infants:ti,ab,kw,de OR newborn:ti,ab,kw,de OR newborns:ti,ab,kw,de OR neonate:ti,ab,kw,de OR neonates:ti,ab,kw,de OR paediatrics OR paediatric
Risk factors	'risk factor'/exp OR 'risk'/exp OR 'risk factor' OR 'risk factors' OR determinant OR determinants OR predictor OR predictors OR 'predictive value' OR risk OR risks
Hypoxemia	'hypoxia'/exp OR 'oxygen saturation'/exp OR hypoxemia OR hypoxaemia OR hypoxia OR 'oxygen saturation'

Mortality	'mortality'/exp OR 'death'/exp OR mortality:ti,ab,kw,de,lnk OR deaths OR fatality OR fatal
LMIC	'developing country':ab,ti OR 'developing countries':ab,ti OR 'developing nation':ab,ti OR 'developing nations':ab,ti OR 'developing population':ab,ti OR 'developing populations':ab,ti OR 'developing world':ab,ti OR 'less developed country':ab,ti OR 'less developed countries':ab,ti OR 'less developed nation':ab,ti OR 'less developed nations':ab,ti OR 'less developed population':ab,ti OR 'less developed populations':ab,ti OR 'less developed world':ab,ti OR 'lesser developed country':ab,ti OR 'lesser developed countries':ab,ti OR 'lesser developed nation':ab,ti OR 'lesser developed nations':ab,ti OR 'lesser developed population':ab,ti OR 'lesser developed populations':ab,ti OR 'lesser developed world':ab,ti OR 'under developed country':ab,ti OR 'under developed countries':ab,ti OR 'under developed nation':ab,ti OR 'under developed nations':ab,ti OR 'under developed population':ab,ti OR 'under developed populations':ab,ti OR 'under developed world':ab,ti OR 'underdeveloped country':ab,ti OR 'underdeveloped countries':ab,ti OR 'underdeveloped nation':ab,ti OR 'underdeveloped nations':ab,ti OR 'underdeveloped population':ab,ti OR 'underdeveloped populations':ab,ti OR 'underdeveloped world':ab,ti OR 'middle income country':ab,ti OR 'middle income countries':ab,ti OR 'middle income nation':ab,ti OR 'middle income nations':ab,ti OR 'middle income population':ab,ti OR 'middle income populations':ab,ti OR 'low income country':ab,ti OR 'low income countries':ab,ti OR 'low income nation':ab,ti OR 'low income nations':ab,ti OR 'low income population':ab,ti OR 'low income populations':ab,ti OR 'lower income country':ab,ti OR 'lower income countries':ab,ti OR 'lower income nation':ab,ti OR 'lower income nations':ab,ti OR 'lower income population':ab,ti OR 'lower income populations':ab,ti OR 'underserved country':ab,ti OR 'underserved countries':ab,ti OR 'underserved nation':ab,ti OR 'underserved nations':ab,ti OR 'underserved population':ab,ti OR 'underserved populations':ab,ti OR 'underserved world':ab,ti OR 'under served country':ab,ti OR 'under served countries':ab,ti OR 'under served nation':ab,ti OR 'under served nations':ab,ti OR 'under served population':ab,ti OR 'under served populations':ab,ti OR 'under served world':ab,ti OR 'deprived country':ab,ti OR 'deprived countries':ab,ti OR 'deprived nation':ab,ti OR 'deprived nations':ab,ti OR 'deprived population':ab,ti OR 'deprived populations':ab,ti OR 'deprived world':ab,ti OR 'poor country':ab,ti OR 'poor countries':ab,ti OR 'poor nation':ab,ti OR 'poor nations':ab,ti OR 'poor population':ab,ti OR 'poor populations':ab,ti OR 'poor world':ab,ti OR 'poorer country':ab,ti OR 'poorer countries':ab,ti OR 'poorer nation':ab,ti OR 'poorer nations':ab,ti OR 'poorer population':ab,ti OR 'poorer populations':ab,ti OR 'poorer world':ab,ti OR 'developing economy':ab,ti OR 'developing economies':ab,ti OR 'less developed economy':ab,ti OR 'less developed economies':ab,ti OR 'lesser developed economy':ab,ti OR 'lesser developed economies':ab,ti OR 'under developed economy':ab,ti OR 'under developed economies':ab,ti OR 'underdeveloped economy':ab,ti OR 'underdeveloped economies':ab,ti OR 'middle income economy':ab,ti OR 'middle income economies':ab,ti OR 'low income economy':ab,ti OR 'low income economies':ab,ti OR 'lower income economy':ab,ti OR 'lower

	income economies':ab,ti OR 'low gdp':ab,ti OR 'low gnp':ab,ti OR 'low gross domestic':ab,ti OR 'low gross national':ab,ti OR 'lower gdp':ab,ti OR 'lower gnp':ab,ti OR 'lower gross domestic':ab,ti OR 'lower gross national':ab,ti OR lmic:ab,ti OR lmics:ab,ti OR 'third world':ab,ti OR 'lami country':ab,ti OR 'lami countries':ab,ti OR 'transitional country':ab,ti OR 'transitional countries':ab,ti OR africa:ti,ab OR asia:ti,ab OR caribbean:ti,ab OR 'west indies':ti,ab OR 'south america':ti,ab OR 'latin america':ti,ab OR 'central america':ti,ab OR 'atlantic islands':ab,ti OR 'commonwealth of independent states':ab,ti OR 'pacific islands':ab,ti OR 'indian ocean islands':ab,ti OR 'eastern europe':ab,ti OR afghanistan:ti,ab OR albania:ti,ab OR algeria:ti,ab OR 'american samoa':ti,ab OR angola:ti,ab OR antigua:ti,ab OR barbuda:ti,ab OR argentina:ti,ab OR armenia:ti,ab OR armenian:ti,ab OR aruba:ti,ab OR azerbaijan:ti,ab OR bahrain:ti,ab OR bangladesh:ti,ab OR barbados:ti,ab OR benin:ti,ab OR byelarus:ti,ab OR byelorussian:ti,ab OR belarus:ti,ab OR belorussian:ti,ab OR belorussia:ti,ab OR belize:ti,ab OR bhutan:ti,ab OR bolivia:ti,ab OR bosnia:ti,ab OR herzegovina:ti,ab OR hercegovina:ti,ab OR botswana:ti,ab OR brasil:ti,ab OR brazil:ti,ab OR bulgaria:ti,ab OR 'burkina faso':ti,ab OR 'burkina fasso':ti,ab OR 'upper volta':ti,ab OR burundi:ti,ab OR urundi:ti,ab OR cambodia:ti,ab OR 'khmer republic':ti,ab OR kampuchea:ti,ab OR cameroon:ti,ab OR cameroons:ti,ab OR cameron:ti,ab OR camerons:ti,ab OR 'cape verde':ti,ab OR 'cabo verde':ti,ab OR 'central african republic':ti,ab OR chad:ti,ab OR chile:ti,ab OR china:ti,ab OR colombia:ti,ab OR comoros:ti,ab OR 'comoro islands':ti,ab OR comores:ti,ab OR mayotte:ti,ab OR congo:ti,ab OR zaire:ti,ab OR 'costa rica':ti,ab OR 'cote d`ivoire' OR 'ivory coast':ti,ab OR croatia:ti,ab OR cuba:ti,ab OR cyprus:ti,ab OR czechoslovakia:ti,ab OR 'czech republic':ti,ab OR slovakia:ti,ab OR 'slovak republic':ti,ab OR djibouti:ti,ab OR 'french somaliland':ti,ab OR dominica:ti,ab OR 'dominican republic':ti,ab OR 'east timor':ti,ab OR 'east timur':ti,ab OR 'timor leste':ti,ab OR ecuador:ti,ab OR egypt:ti,ab OR 'united arab republic':ti,ab OR 'el salvador':ti,ab OR eritrea:ti,ab OR estonia:ti,ab OR eswatini:ti,ab OR ethiopia:ti,ab OR fiji:ti,ab OR gabon:ti,ab OR 'gabonese republic':ti,ab OR gambia:ti,ab OR gaza:ti,ab OR 'georgia republic':ti,ab OR 'georgian republic':ti,ab OR ghana:ti,ab OR 'gold coast':ti,ab OR grenada:ti,ab OR guatemala:ti,ab OR guinea:ti,ab OR guam:ti,ab OR guiana:ti,ab OR guyana:ti,ab OR haiti:ti,ab OR honduras:ti,ab OR india:ti,ab OR maldives:ti,ab OR indonesia:ti,ab OR iran:ti,ab OR iraq:ti,ab OR 'isle of man':ti,ab OR jamaica:ti,ab OR jordan:ti,ab OR kazakhstan:ti,ab OR kazakh:ti,ab OR kenya:ti,ab OR kiribati:ti,ab OR korea:ti,ab OR kosovo:ti,ab OR kyrgyzstan:ti,ab OR kirghizia:ti,ab OR 'kyrgyz republic':ti,ab OR kirghiz:ti,ab OR kirgizstan:ti,ab OR 'lao pdr':ti,ab OR laos:ti,ab OR latvia:ti,ab OR lebanon:ti,ab OR lesotho:ti,ab OR basutoland:ti,ab OR liberia:ti,ab OR libya:ti,ab OR lithuania:ti,ab OR macedonia:ti,ab OR madagascar:ti,ab OR 'malagasy republic':ti,ab OR malaysia:ti,ab OR malaya:ti,ab OR malay:ti,ab OR sabah:ti,ab OR sarawak:ti,ab OR malawi:ti,ab OR nyasaland:ti,ab OR mali:ti,ab OR malta:ti,ab OR 'marshall islands':ti,ab OR mauritania:ti,ab OR mauritius:ti,ab OR melanesia:ab,ti OR 'agalega islands':ti,ab OR mexico:ti,ab OR micronesia:ti,ab OR 'middle east':ti,ab OR moldova:ti,ab OR moldovia:ti,ab OR moldovian:ti,ab

	OR mongolia:ti,ab OR morocco:ti,ab OR ifni:ti,ab OR mozambique:ti,ab OR myanmar:ti,ab OR myanma:ti,ab OR burma:ti,ab OR namibia:ti,ab OR nepal:ti,ab OR 'netherlands antilles':ti,ab OR 'new caledonia':ti,ab OR nicaragua:ti,ab OR niger:ti,ab OR nigeria:ti,ab OR 'northern mariana islands':ti,ab OR oman:ti,ab OR muscat:ti,ab OR pakistan:ti,ab OR palau:ti,ab OR palestine:ti,ab OR panama:ti,ab OR paraguay:ti,ab OR peru:ti,ab OR philippines:ti,ab OR philipines:ti,ab OR phillipines:ti,ab OR phillippines:ti,ab OR poland:ti,ab OR portugal:ti,ab OR 'puerto rico':ti,ab OR romania:ti,ab OR rumania:ti,ab OR roumania:ti,ab OR russia:ti,ab OR russian:ti,ab OR rwanda:ti,ab OR ruanda:ti,ab OR 'saint kitts':ti,ab OR 'st kitts':ti,ab OR nevis:ti,ab OR 'saint lucia':ti,ab OR 'st lucia':ti,ab OR 'saint vincent':ti,ab OR 'st vincent':ti,ab OR grenadines:ti,ab OR samoa:ti,ab OR 'samoan islands':ti,ab OR 'navigator island':ti,ab OR 'navigator islands':ti,ab OR 'sao tome':ti,ab OR 'saudi arabia':ti,ab OR senegal:ti,ab OR serbia:ti,ab OR montenegro:ti,ab OR seychelles:ti,ab OR 'sierra leone':ti,ab OR slovenia:ti,ab OR 'sri lanka':ti,ab OR ceylon:ti,ab OR 'solomon islands':ti,ab OR somalia:ti,ab OR sudan:ti,ab OR suriname:ti,ab OR surinam:ti,ab OR swaziland:ti,ab OR syria:ti,ab OR syrian:ti,ab OR tajikistan:ti,ab OR tadzhikistan:ti,ab OR tadjikistan:ti,ab OR tadzhik:ti,ab OR tanzania:ti,ab OR thailand:ti,ab OR togo:ti,ab OR 'togolese republic':ti,ab OR tonga:ti,ab OR trinidad:ti,ab OR tobago:ti,ab OR tunisia:ti,ab OR turkey:ti,ab OR turkmenistan:ti,ab OR turkmen:ti,ab OR tuvalu:ti,ab OR uganda:ti,ab OR ukraine:ti,ab OR uruguay:ti,ab OR ussr:ti,ab OR 'soviet union':ti,ab OR 'union of soviet socialist republics':ti,ab OR uzbekistan:ti,ab OR uzbek OR vanuatu:ti,ab OR 'new hebrides':ti,ab OR venezuela:ti,ab OR vietnam:ti,ab OR 'viet nam':ti,ab OR 'west bank':ti,ab OR yemen:ti,ab OR yugoslavia:ti,ab OR zambia:ti,ab OR zimbabwe:ti,ab OR rhodesia:ti,ab OR 'developing country'/exp OR 'africa'/de OR 'africa south of the sahara'/de OR 'north africa'/de OR 'central africa'/de OR 'asia'/de OR 'south asia'/de OR 'southeast asia'/de OR 'south america'/de OR 'central america'/de OR 'south and central america'/de OR 'atlantic islands'/de OR 'caribbean islands'/de OR 'pacific islands'/de OR 'indian ocean'/de OR 'eastern europe'/de OR 'afghanistan'/exp OR 'albania'/exp OR 'algeria'/exp OR 'american samoa'/exp OR 'angola'/exp OR 'antigua and barbuda'/exp OR 'argentina'/exp OR 'armenia'/exp OR 'azerbaijan'/exp OR 'bahrain'/exp OR 'bangladesh'/exp OR 'barbados'/exp OR 'benin'/exp OR 'belarus'/exp OR 'baltic states'/exp OR 'belize'/exp OR 'bhutan'/exp OR 'bolivia'/exp OR 'bosnia and herzegovina'/exp OR 'botswana'/exp OR 'brazil'/exp OR 'bulgaria'/exp OR 'burkina faso'/exp OR 'burundi'/exp OR 'cambodia'/exp OR 'cameroon'/exp OR 'cape verde'/exp OR 'central african republic'/exp OR 'chad'/exp OR 'chile'/exp OR 'china'/exp OR 'colombia'/exp OR 'comoros'/exp OR 'congo'/exp OR 'costa rica'/exp OR 'cote d\`ivoire'/exp OR 'croatia'/exp OR 'cuba'/exp OR 'cyprus'/exp OR 'czechoslovakia'/exp OR 'czech republic'/exp OR 'slovakia'/exp OR 'djibouti'/exp OR 'democratic republic congo'/exp OR 'dominica'/exp OR 'dominican republic'/exp OR 'timor-leste'/exp OR 'ecuador'/exp OR 'egypt'/exp OR 'el salvador'/exp OR 'eritrea'/exp OR 'estonia'/exp OR 'eswatini'/exp OR 'ethiopia'/exp OR 'french guiana'/exp OR 'fiji'/exp OR 'gabon'/exp OR 'gambia'/exp OR 'georgia (republic)'/exp OR

	'ghana'/exp OR 'grenada'/exp OR 'guatemala'/exp OR 'guinea'/exp OR 'guinea bissau'/exp OR 'guam'/exp OR 'guyana'/exp OR 'haiti'/exp OR 'honduras'/exp OR 'india'/exp OR 'indonesia'/exp OR 'iran'/exp OR 'iraq'/exp OR 'jamaica'/exp OR 'jordan'/exp OR 'kazakhstan'/exp OR 'kenya'/exp OR 'korea'/exp OR 'kyrgyzstan'/exp OR 'laos'/exp OR 'latvia'/exp OR 'lebanon'/exp OR 'lesotho'/exp OR 'liberia'/exp OR 'libyan arab jamahiriya'/exp OR 'lithuania'/exp OR 'macedonia (republic)'/exp OR 'madagascar'/exp OR 'malaysia'/exp OR 'malawi'/exp OR 'mali'/exp OR 'malta'/exp OR 'mauritania'/exp OR 'mauritius'/exp OR 'melanesia'/exp OR 'mexico'/exp OR 'federated states of micronesia'/exp OR 'middle east'/de OR 'moldova'/exp OR 'mongolia'/exp OR 'montenegro'/exp OR 'morocco'/exp OR 'mozambique'/exp OR 'myanmar'/exp OR 'namibia'/exp OR 'nepal'/exp OR 'netherlands antilles'/exp OR 'new caledonia'/exp OR 'nicaragua'/exp OR 'niger'/exp OR 'nigeria'/exp OR 'north korea'/exp OR 'oman'/exp OR 'pakistan'/exp OR 'palau'/exp OR 'panama'/exp OR 'papua new guinea'/exp OR 'paraguay'/exp OR 'peru'/exp OR 'philippines'/exp OR 'poland'/exp OR 'portugal'/exp OR 'puerto rico'/exp OR 'romania'/exp OR 'russian federation'/exp OR 'rwanda'/exp OR 'saint kitts and nevis'/exp OR 'saint lucia'/exp OR 'saint vincent and the grenadines'/exp OR 'samoan islands'/exp OR 'samoa'/exp OR 'saudi arabia'/exp OR 'senegal'/exp OR 'serbia'/exp OR 'montenegro (republic)'/exp OR 'seychelles'/exp OR 'sierra leone'/exp OR 'slovenia'/exp OR 'sri lanka'/exp OR 'somalia'/exp OR 'south korea'/exp OR 'south africa'/exp OR 'sudan'/exp OR 'suriname'/exp OR 'swaziland'/exp OR 'syrian arab republic'/exp OR 'tajikistan'/exp OR 'tanzania'/exp OR 'thailand'/exp OR 'togo'/exp OR 'tonga'/exp OR 'trinidad and tobago'/exp OR 'tunisia'/exp OR 'turkey (republic)'/exp OR 'turkmenistan'/exp OR 'uganda'/exp OR 'ukraine'/exp OR 'uruguay'/exp OR 'ussr'/exp OR 'uzbekistan'/exp OR 'vanuatu'/exp OR 'venezuela'/exp OR 'viet nam'/exp OR 'yemen'/exp OR 'yugoslavia'/exp OR 'yugoslavia (pre-1992)'/exp OR 'zambia'/exp OR 'zimbabwe'/exp

Scopus

Pneumonia	TITLE-ABS-KEY (Pneumonia OR "respiratory infection" OR "respiratory infections" OR "lower respiratory tract infection*" OR bronchiolitis)
Children	TITLE-ABS-KEY (child OR children OR pediatrics OR pediatric OR paediatrics OR paediatric OR infant OR infants OR newborn OR newborns OR neonate OR neonates)
Risk factors	TITLE-ABS-KEY ("risk factor" OR "risk factors" OR determinant OR determinants OR predictor OR predictors OR "predictive value" OR risk OR risks)
Hypoxemia	TITLE-ABS-KEY (hypoxemia OR hypoxaemia OR hypoxia OR "oxygen saturation")
Mortality	TITLE-ABS-KEY (mortality OR death OR deaths OR fatality OR fatal)

Coding: (TITLE-ABS-KEY((Pneumonia OR "respiratory infection" OR "respiratory infections" OR "lower respiratory tract infection*" OR bronchiolitis))) AND (TITLE-ABS-KEY (child OR children OR pediatrics OR pediatric OR

paediatrics OR paediatric OR infant OR infants OR newborn OR newborns OR neonate OR neonates)) AND (TITLE-ABS-KEY ("risk factor" OR "risk factors" OR determinant OR determinants OR predictor OR predictors OR "predictive value" OR risk OR risks)) AND (TITLE-ABS-KEY (mortality OR death OR deaths OR fatality OR fatal)) AND (TITLE-ABS-KEY("Antigua and Barbuda " OR " Atlantic Islands " OR " Baltic States " OR " Commonwealth of Independent States " OR " Democratic People's Republic of Korea " OR " Democratic Republic of the Congo " OR " deprived countries " OR " deprived population " OR " deprived populations " OR " developing countries " OR " developing country " OR " developing economies " OR " developing economy " OR " developing nation " OR " developing nations " OR " developing population " OR " developing populations " OR " developing world " OR " Equatorial Guinea " OR " French Guiana " OR " Georgia Republic " OR " Independent State of Samoa " OR " Indian Ocean Islands " OR " lami countries " OR " lami country " OR " less developed countries " OR " less developed country " OR " less developed economies " OR " less developed economy " OR " less developed nation " OR " less developed nations " OR " less developed world " OR " lesser developed countries " OR " lesser developed nations " OR " low gdp " OR " low gnp " OR " low gross domestic " OR " low gross national " OR " low income countries " OR " low income country " OR " low income economies " OR " low income economy " OR " low income nations " OR " low income population " OR " low income populations " OR " lower gdp " OR " lower gross domestic " OR " lower income countries " OR " lower income country " OR " lower income nations " OR " lower income population " OR " lower income populations " OR " Macedonia Republic " OR " Melanesia " OR " middle income countries " OR " middle income country " OR " middle income economies " OR " middle income nation " OR " middle income nations " OR " middle income population " OR " middle income populations " OR " Pacific Islands " OR " poor countries " OR " poor country " OR " poor nation " OR " poor nations " OR " poor population " OR " poor populations " OR " poor world " OR " poorer countries " OR " poorer nations " OR " poorer population " OR " poorer populations " OR " Republic of Belarus " OR " Saint Kitts and Nevis " OR " Saint Vincent and the Grenadines " OR " South Sudan " OR " third world " OR " transitional countries " OR " transitional country " OR " Trinidad and Tobago " OR " under developed countries " OR " under developed country " OR " under developed nations " OR " under developed world " OR " under served population " OR " under served populations " OR " underdeveloped countries " OR " underdeveloped country " OR " underdeveloped economies " OR " underdeveloped nations " OR " underdeveloped population " OR " underdeveloped world " OR " underserved countries " OR " underserved nations " OR " underserved population " OR " underserved populations " OR " Afghanistan " OR " Africa " OR " Albania " OR " Algeria " OR " American Samoa " OR " Angola " OR " Argentina " OR " Armenia " OR " Asia " OR " Azerbaijan " OR " Bahrain " OR " Bangladesh " OR " Barbados " OR " Belize " OR " Benin " OR " Bhutan " OR " Bolivia " OR " Bosnia-Herzegovina " OR " Botswana " OR " Brazil " OR " Bulgaria " OR " Burkina Faso " OR " Burundi " OR " Cambodia " OR " Cameroon " OR " Cape Verde " OR " Caribbean Region " OR " Central African Republic " OR " Central America " OR " Chad " OR " Chile " OR " China " OR " Colombia " OR " Comoros " OR " Congo " OR " Costa Rica " OR " Cote d'Ivoire " OR " Croatia " OR " Cuba " OR " Cyprus " OR " Czech Republic " OR " Czechoslovakia " OR " Developing Countries " OR " Djibouti " OR " Dominica " OR " Dominican Republic " OR " East Timor " OR " Ecuador " OR " Egypt " OR " El Salvador " OR " Eritrea " OR " Estonia " OR " Ethiopia " OR " Fiji " OR " Gabon " OR " Gambia " OR " Ghana " OR " Greece " OR " Grenada " OR " Guam " OR " Guatemala " OR " Guinea " OR " Guinea-Bissau " OR " Guyana " OR " Haiti " OR " Honduras " OR " Hungary " OR " India " OR " Indonesia " OR " Iran " OR " Iraq " OR " Jamaica " OR " Jordan " OR " Kazakhstan " OR " Kenya " OR " Korea " OR " Kyrgyzstan " OR " Laos " OR " Latin America " OR " Latvia " OR " Lebanon " OR " Lesotho " OR " Liberia " OR " Libya " OR " Lithuania " OR " lmic " OR " lmics " OR " Madagascar " OR " Malawi " OR " Malaysia " OR " Mali " OR " Malta " OR " Mauritania " OR " Mauritius " OR " Mexico " OR " Micronesia " OR " Middle East " OR " Moldova " OR " Mongolia " OR " Montenegro " OR " Montenegro " OR " Morocco " OR " Mozambique " OR " Myanmar " OR " Namibia " OR " Nepal " OR " Netherlands Antilles " OR " New Caledonia " OR " Nicaragua " OR " Niger " OR " Nigeria " OR " Oman " OR " Pakistan " OR " Palau " OR " Panama " OR " Papua New Guinea " OR " Paraguay " OR " Peru " OR " Philippines " OR " Poland " OR " Portugal " OR " Puerto Rico " OR " Romania " OR " Russia " OR " Rwanda " OR " Saint Lucia " OR " Samoa " OR " Saudi Arabia " OR " Senegal " OR " Serbia " OR " Seychelles " OR " Sierra Leone " OR " Slovakia " OR " Slovenia " OR " Somalia " OR " South Africa " OR " South America " OR " Sri Lanka " OR " Sudan " OR " Suriname " OR " Swaziland " OR " Syria " OR " Tajikistan " OR " Tanzania " OR " Thailand " OR " Togo " OR " Tonga " OR " Tunisia " OR " Turkey " OR " Turkmenistan " OR " Uganda " OR " Ukraine " OR " Uruguay " OR " USSR " OR " Uzbekistan " OR " Vanuatu " OR " Venezuela " OR " Vietnam " OR " West Indies " OR " Yemen " OR " Yugoslavia " OR " Zambia " OR " Zimbabwe")) AND PUBYEAR > 2022 AND PUBYEAR < 2026

Web of Science

Pneumonia	TS=(Pneumonia OR "respiratory infection" OR "respiratory infections" OR "lower respiratory tract infection*" OR bronchiolitis)
Children	TS=(child OR children OR pediatrics OR pediatric OR paediatrics OR paediatric OR infant OR infants OR newborn OR newborns OR neonate OR neonates)

Risk factors	TS=("risk factor" OR "risk factors" OR determinant OR determinants OR predictor OR predictors OR "predictive value" OR risk OR risks)
Hypoxemia	TS=(hypoxemia OR hypoxaemia OR hypoxia OR "oxygen saturation")
Mortality	TS=(mortality OR death OR deaths OR fatality OR fatal)
LMIC	TS=("Antigua and Barbuda " OR " Atlantic Islands " OR " Baltic States " OR " Commonwealth of Independent States " OR " Democratic People's Republic of Korea " OR " Democratic Republic of the Congo " OR " deprived countries " OR " deprived population " OR " deprived populations " OR " developing countries " OR " developing country " OR " developing economies " OR " developing economy " OR " developing nation " OR " developing nations " OR " developing population " OR " developing populations " OR " developing world " OR " Equatorial Guinea " OR " French Guiana " OR " Georgia Republic " OR " Independent State of Samoa " OR " Indian Ocean Islands " OR " lami countries " OR " lami country " OR " less developed countries " OR " less developed country " OR " less developed economies " OR " less developed economy " OR " less developed nation " OR " less developed nations " OR " less developed world " OR " lesser developed countries " OR " lesser developed nations " OR " low gdp " OR " low gnp " OR " low gross domestic " OR " low gross national " OR " low income countries " OR " low income country " OR " low income economies " OR " low income economy " OR " low income nations " OR " low income population " OR " low income populations " OR " lower gdp " OR " lower gross domestic " OR " lower income countries " OR " lower income country " OR " lower income nations " OR " lower income population " OR " lower income populations " OR " Macedonia Republic " OR " Melanesia " OR " middle income countries " OR " middle income country " OR " middle income economies " OR " middle income nation " OR " middle income nations " OR " middle income population " OR " middle income populations " OR " Pacific Islands " OR " poor countries " OR " poor country " OR " poor nation " OR " poor nations " OR " poor population " OR " poor populations " OR " poor world " OR " poorer countries " OR " poorer nations " OR " poorer population " OR " poorer populations " OR " Republic of Belarus " OR " Saint Kitts and Nevis " OR " Saint Vincent and the Grenadines " OR " South Sudan " OR " third world " OR " transitional countries " OR " transitional country " OR " Trinidad and Tobago " OR " under developed countries " OR " under developed country " OR " under developed nations " OR " under developed world " OR " under served population " OR " under served populations " OR " underdeveloped countries " OR " underdeveloped country " OR " underdeveloped economies " OR " underdeveloped nations " OR " underdeveloped population " OR " underdeveloped world " OR " underserved countries " OR " underserved nations " OR " underserved population " OR " underserved populations " OR " Afghanistan " OR " Africa " OR " Albania " OR " Algeria " OR " American Samoa " OR " Angola " OR " Argentina " OR " Armenia " OR " Asia " OR " Azerbaijan " OR " Bahrain " OR " Bangladesh " OR " Barbados " OR " Belize " OR " Benin " OR " Bhutan " OR " Bolivia " OR " Bosnia-Herzegovina " OR " Botswana " OR " Brazil " OR " Bulgaria " OR " Burkina Faso " OR " Burundi " OR " Cambodia " OR " Cameroon " OR " Cape Verde " OR " Caribbean Region " OR " Central

	African Republic " OR " Central America " OR " Chad " OR " Chile " OR " China " OR " Colombia " OR " Comoros " OR " Congo " OR " Costa Rica " OR " Cote d'Ivoire " OR " Croatia " OR " Cuba " OR " Cyprus " OR " Czech Republic " OR " Czechoslovakia " OR " Developing Countries " OR " Djibouti " OR " Dominica " OR " Dominican Republic " OR " East Timor " OR " Ecuador " OR " Egypt " OR " El Salvador " OR " Eritrea " OR " Estonia " OR " Ethiopia " OR " Fiji " OR " Gabon " OR " Gambia " OR " Ghana " OR " Greece " OR " Grenada " OR " Guam " OR " Guatemala " OR " Guinea " OR " Guinea-Bissau " OR " Guyana " OR " Haiti " OR " Honduras " OR " Hungary " OR " India " OR " Indonesia " OR " Iran " OR " Iraq " OR " Jamaica " OR " Jordan " OR " Kazakhstan " OR " Kenya " OR " Korea " OR " Kyrgyzstan " OR " Laos " OR " Latin America " OR " Latvia " OR " Lebanon " OR " Lesotho " OR " Liberia " OR " Libya " OR " Lithuania " OR " lmic " OR " lmics " OR " Madagascar " OR " Malawi " OR " Malaysia " OR " Mali " OR " Malta " OR " Mauritania " OR " Mauritius " OR " Mexico " OR " Micronesia " OR " Middle East " OR " Moldova " OR " Mongolia " OR " Montenegro " OR " Montenegro " OR " Morocco " OR " Mozambique " OR " Myanmar " OR " Namibia " OR " Nepal " OR " Netherlands Antilles " OR " New Caledonia " OR " Nicaragua " OR " Niger " OR " Nigeria " OR " Oman " OR " Pakistan " OR " Palau " OR " Panama " OR " Papua New Guinea " OR " Paraguay " OR " Peru " OR " Philippines " OR " Poland " OR " Portugal " OR " Puerto Rico " OR " Romania " OR " Russia " OR " Rwanda " OR " Saint Lucia " OR " Samoa " OR " Saudi Arabia " OR " Senegal " OR " Serbia " OR " Seychelles " OR " Sierra Leone " OR " Slovakia " OR " Slovenia " OR " Somalia " OR " South Africa " OR " South America " OR " Sri Lanka " OR " Sudan " OR " Suriname " OR " Swaziland " OR " Syria " OR " Tajikistan " OR " Tanzania " OR " Thailand " OR " Togo " OR " Tonga " OR " Tunisia " OR " Turkey " OR " Turkmenistan " OR " Uganda " OR " Ukraine " OR " Uruguay " OR " USSR " OR " Uzbekistan " OR " Vanuatu " OR " Venezuela " OR " Vietnam " OR " West Indies " OR " Yemen " OR " Yugoslavia " OR " Zambia " OR " Zimbabwe")

Global Index Medicus (advanced search: title, abstract, subject)

Pneumonia	C01.748.610$ OR C08.381.677$ OR C08.730.610$ OR C01.748.099.135$ OR C08.127.446.135$ OR C08.381.495.146.135$ OR C08.730.099.135$<br><br>OR Pneumonia OR "respiratory infection" OR "respiratory infections" OR "lower respiratory tract infection" OR "lower respiratory tract infections" OR bronchiolitis
Children	M01.060.406$ OR M01.060.703$ OR H02.403.670$<br><br>OR child OR children OR pediatrics OR pediatric OR paediatrics OR paediatric OR infant OR infants OR newborn OR newborns OR neonate OR neonates
Risk factors	E05.318.740.600.800.725$ OR N05.715.350.200.700$ OR N05.715.360.750.625.700.700$ OR N06.850.490.625.750$ OR N06.850.520.830.600.800.725$ OR SP5.312.109.198.566.458 OR E05.318.740.600.800$ G17.680.750$ OR N05.715.360.750.625.700$ OR

	N06.850.520.830.600.800$ OR "risk factor" OR "risk factors" OR determinant OR determinants OR predictor OR predictors OR "predictive value" OR risk OR risks
Hypoxemia	C23.888.852.079$ OR hypoxemia OR hypoxaemia OR hypoxia OR "oxygen saturation"
Mortality	E05.318.308.985.550$ OR N01.224.935.698$ OR N06.850.505.400.975.550$ OR N06.850.520.308.985.550$ OR SP3.311.300.392.432$ OR SP5.467.413.330.903$ OR C23.550.260$ OR mortality OR death OR deaths OR fatality OR fatal

LILACS

Pneumonia	C01.748.610$ OR C08.381.677$ OR C08.730.610$ OR C01.748.099.135$ OR C08.127.446.135$ OR C08.381.495.146.135$ OR C08.730.099.135$<br>OR Pneumonia OR "respiratory infection" OR "respiratory infections" OR "lower respiratory tract infection*" OR bronchiolitis
Children	M01.060.406$ OR M01.060.703$ OR H02.403.670$<br>OR child OR children OR pediatrics OR pediatric OR paediatrics OR paediatric OR infant OR infants OR newborn OR newborns OR neonate OR neonates
Risk factors	E05.318.740.600.800.725$ OR N05.715.350.200.700$ OR N05.715.360.750.625.700.700$ OR N06.850.490.625.750$ OR N06.850.520.830.600.800.725$ OR SP5.312.109.198.566.458 OR E05.318.740.600.800$ G17.680.750$ OR N05.715.360.750.625.700$ OR N06.850.520.830.600.800$ OR "risk factor" OR "risk factors" OR determinant OR determinants OR predictor OR predictors OR "predictive value" OR risk OR risks
Hypoxemia	C23.888.852.079$ OR hypoxemia OR hypoxaemia OR hypoxia OR "oxygen saturation"
Mortality	E05.318.308.985.550$ OR N01.224.935.698$ OR N06.850.505.400.975.550$ OR N06.850.520.308.985.550$ OR SP3.311.300.392.432$ OR SP5.467.413.330.903$ OR C23.550.260$ OR mortality OR death OR deaths OR fatality OR fatal

File S1. Quality In Prognosis Studies (QUIPS) tool

**ASSESSMENT FOR RISK OF BIAS**				
First author	Reviewer........................			
**Biases**	**Issues to consider for judging overall rating of "Risk of bias"**	**Study Methods and Comments**	**Rating of reporting**	**Rating of risk of bias**
Assess the risk of each potential bias	These issues will guide your thinking and judgement about the overall risk of bias within each of the 6 domains.	Provide comments or excerpts to facilitate the consensus process that will follow	Y: yes<br>N: no<br>P: partial<br>U: unknown<br>NA: not applicable	HIGH<br>MODERATE<br>LOW<br>UNKNOWN
**1) STUDY PARTICIPATION**	The study sample adequately represents the population of interest			SUMMARY
a. Adequate participation in the study by eligible persons (>80%)				
b. Description of the source population or population of interest				
c. Description of the baseline study sample				
d. Adequate description of the sampling frame and recruitment.				
e. Adequate description of the period and place of recruitment				
f. Adequate description of inclusion and exclusion criteria				
**2) STUDY ATTRITION**	The study data available adequately represent the study sample			SUMMARY
a. Adequate response rate for study participants (> 80%)				
b. Description of attempts to collect information on participants who dropped out				
c. Reasons for loss to follow-up are provided				
d. Adequate description of participants lost to follow-up				
e. There are no important differences between participants who completed the study and who did not				
**3) PROGNOSTIC FACTORS MEASUREMENT**	The PF is measured in a similar way for all participants			SUMMARY
a. A clear definition or description of the PF is provided				
b. Method of PF measurement is adequately valid and reliable (i.e. direct ascertainment; secure record, hospital record)				

c. Continuous variables are reported or appropriate cut points are used			
d. The method and setting of measurement of PF is the same for all study participants			
e. Adequate proportion of the study sample has complete data for the PF *(> 80%)*			
f. Appropriate methods of imputation are used for missing PF data			
**4) OUTCOME MEASUREMENT** The outcome of interest is measured in a similar way for all participants			SUMMARY
a. A clear definition of the outcome of interest is provided (including time of death)			
b. Method of outcome measurement used is adequately valid and reliable (i.e. independent blind assessment, hospital record or record linkage)			
c. The method and setting of outcome measurement is the same for all study participants			
**5) STUDY CONFOUNDING** Important potential confounder are appropriately accounted for			SUMMARY
a. Most important confounders are measured			
b. Clear definitions of the important confounders measured are provided			
c. Measurement of all important confounders is adequately valid and reliable			
d. The method and setting of confounding measurement are the same for all study participants			
e. Appropriate methods are used if imputation is used for missing confounder data			
f. Important potential confounders are accounted for in the study design (by limiting the study to specific population groups, or by matching)			
g. Important potential confounders are accounted for in the analysis *(by stratification, multivariate regression)*			
**6) STATISTICAL ANALYSIS AND PRESENTATION** The statistical analysis is appropriate, and all primary outcomes are reported			SUMMARY
a. Sufficient presentation of data to assess the adequacy of the analytic strategy			
b. Strategy for model building is appropriate and is based on a conceptual framework or model			
c. The selected statistical model is adequate for the design of the study			

d. There is no selective reporting of results (based on the study protocol, if available, or on the method section)			

File S2. Assessment of the Overall Bias for Each Single Study

Number of domains out of the total 6 domains in each category			OVERALL RISK OF BIAS
Low	Moderate/Unknown	High	
6<br><br>4 or 5	0<br><br>1 or 2	0<br><br>0	LOW RISK
3	3<br><br>1	0<br><br>1	MODERATE RISK
	 <br>4 or more	2 or more	HIGH RISK

Table S2. Additional Characteristics of Included Studies

Author, year	Design	Comorbidities	Altitude (meters)	LRI definition
Abdulkadir, 2015	Cross sectional		307	British Thoracic Society Guidelines
Alwadhi, 2017	Cohort		300	WHO
Aroor, 2022	Cohort	Severe acute malnutrition	75	Radiological; Clinical
Asghar, 2008	RCT	Severe acute malnutrition	various (4-2250)	WHO
Awasthi, 2023	Cohort		various (52-197)	WHO
Bassat, 2016	Cohort	HIV infection	50	WHO
Benet, 2017	Cohort	HIV infection; Severe acute malnutrition	various (126-1276)	WHO; Radiological
Bui-Binh-Bao, 2021	Cohort		0	WHO
Chakraborty, 2020	Case control	Severe acute malnutrition	2045	WHO
Chisti, 2011	Cohort	Diarrhea	4	WHO
Chisti, 2013	Case control	Severe acute malnutrition	4	Radiological
Colbourn, 2020	Cohort, data linkage	HIV infection; Severe acute malnutrition	various (1050-1181)	WHO
Dembele, 2019	Case series	Severe acute malnutrition	various (40-300)	WHO
Demers, 2000	Cohort		386	WHO
Dias, 2024	Cohort	Diarrhea	various	Clinical
Djelantik, 2003	Cohort		26	WHO
Duke, 2001	Cohort		1600	WHO with PNG guidelines
Graham, 2011	Cohort	HIV infection	1039	WHO
Graham, 2019	Cohort	HIV infection; Severe acute malnutrition; Diarrhea	various (46-400)	WHO
Hooli, 2020	Cohort	Severe acute malnutrition	various (1,000-1,200)	Malawi Ministry of Health or WHO
Junge, 2006	Cohort		29	Clinical
Kapoor, 2022	Case control		123	WHO
Kuti, 2013	Cohort	Severe acute malnutrition	3	WHO
Mc Nally, 2007	Cohort	HIV infection	22	WHO
McCollum, 2019	RCT	HIV infection; Severe acute malnutrition	476	WHO
McCollum, 2023	Cohort	Severe acute malnutrition	various (17-23)	WHO

Mwaniki, 2009	Cohort		0	WHO
Nantanda, 2008	Cohort		1260	WHO
Nantanda, 2014	Cohort		1260	WHO then South African guidelines
Odeyemi, 2021	Cohort	Severe acute malnutrition	318	WHO; Radiological
Onyango, 1993	Cohort		1670	Clinical
Pathak, 2020	Cohort		412	WHO
Pranathi, 2023	Cohort	Severe acute malnutrition	920	WHO
Rahman, 2021	Cohort	Severe acute malnutrition	32	WHO
Ramakrishna, 2012	Cohort	HIV infection; Severe acute malnutrition	1039	WHO
Reed, 2012	Cohort		1753	Clinical
Rodriguez, 2010	Case control		2640	ICD-10 death certificate for ALRI and clinical records
Shi, 2020	Cohort	Severe acute malnutrition	21	British Thoracic Society Guidelines
Sigauque, 2009	Cohort		50	WHO
Smyth, 1998	Cohort		1150	WHO
Tekam, 2023	Cross sectional	Severe acute malnutrition	500	Unknown
Usen, 1999	Cohort		2	Clinical
Zampoli, 2017	Cohort	HIV infection; Severe acute malnutrition; Diarrhea	42	Clinical
Zhang, 2013	Cohort		4	WHO

Figure S1: Odds of mortality from lower respiratory infection among children comparing hypoxemic versus non-hypoxemic groups, stratified by country Hib and PCV vaccine era.

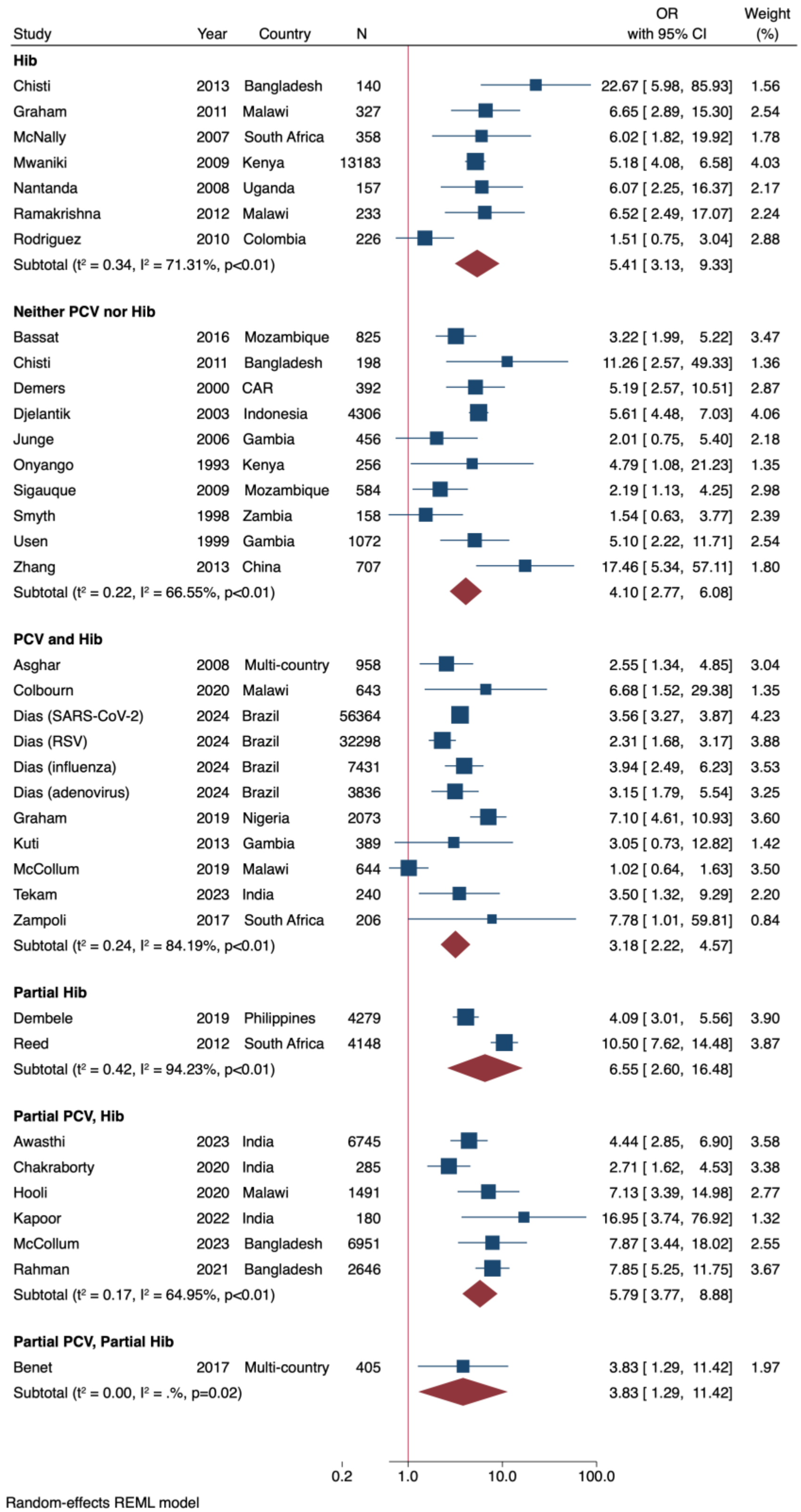

Figure S2: Odds of mortality from lower respiratory infection among children comparing hypoxemic versus non-hypoxemic groups, stratified by study altitude category.

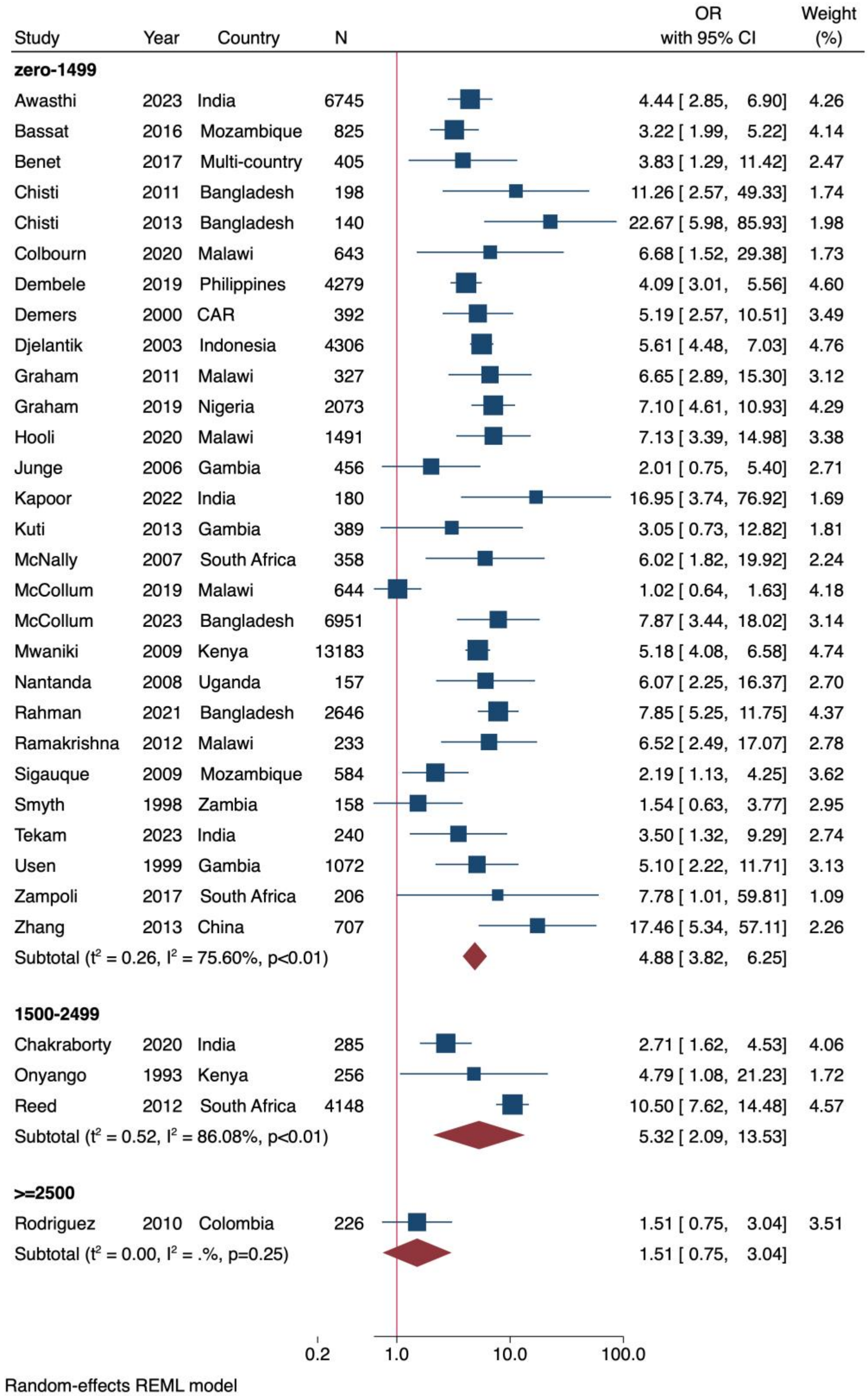

Figure S3: Odds of mortality from lower respiratory infection among children comparing hypoxemic versus non-hypoxemic groups, stratified by study definition of lower respiratory infection

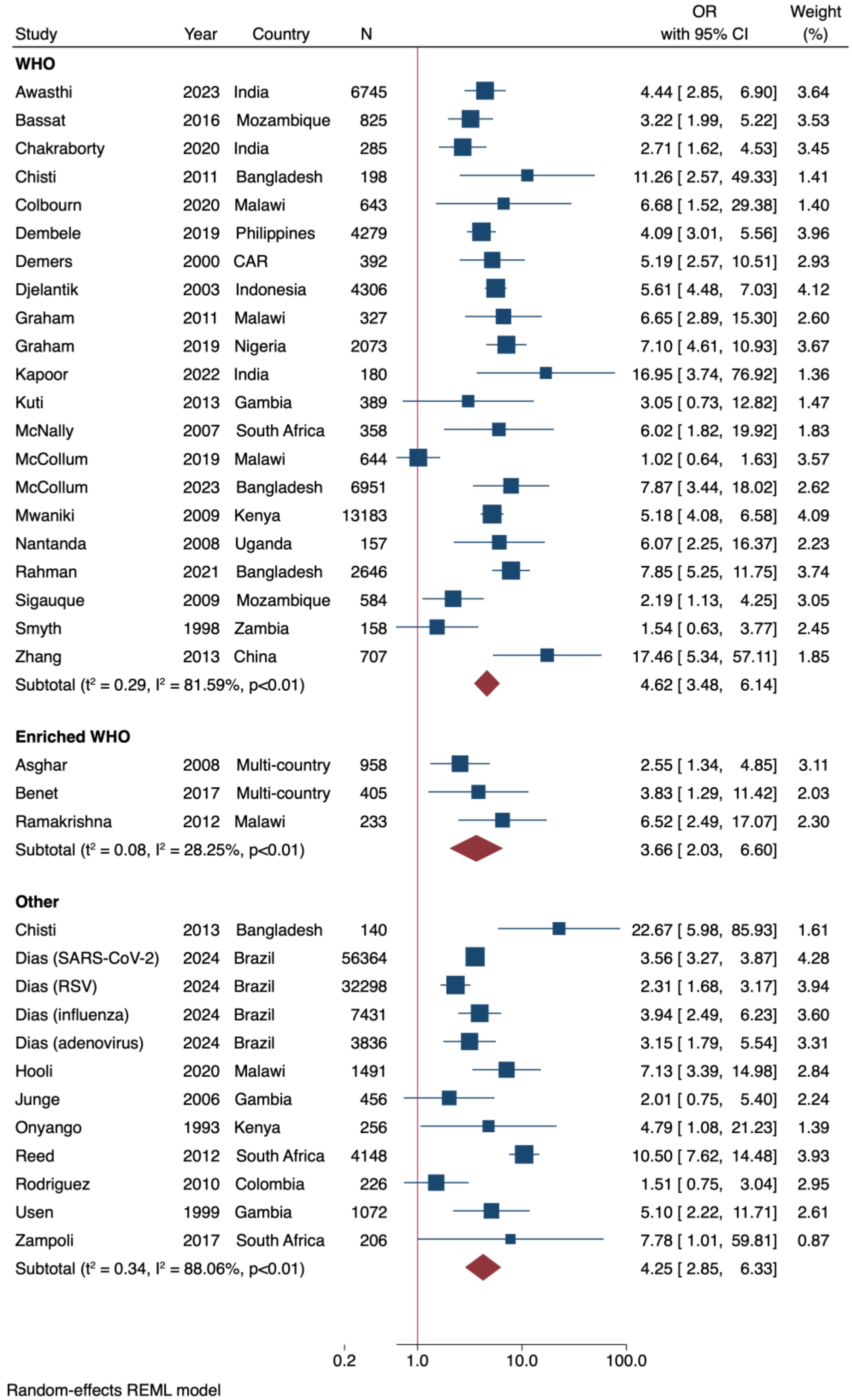

Figure S4: Odds of mortality from lower respiratory infection among children comparing hypoxemic versus non-hypoxemic groups, stratified by study risk of bias (low vs. moderate) and overall.

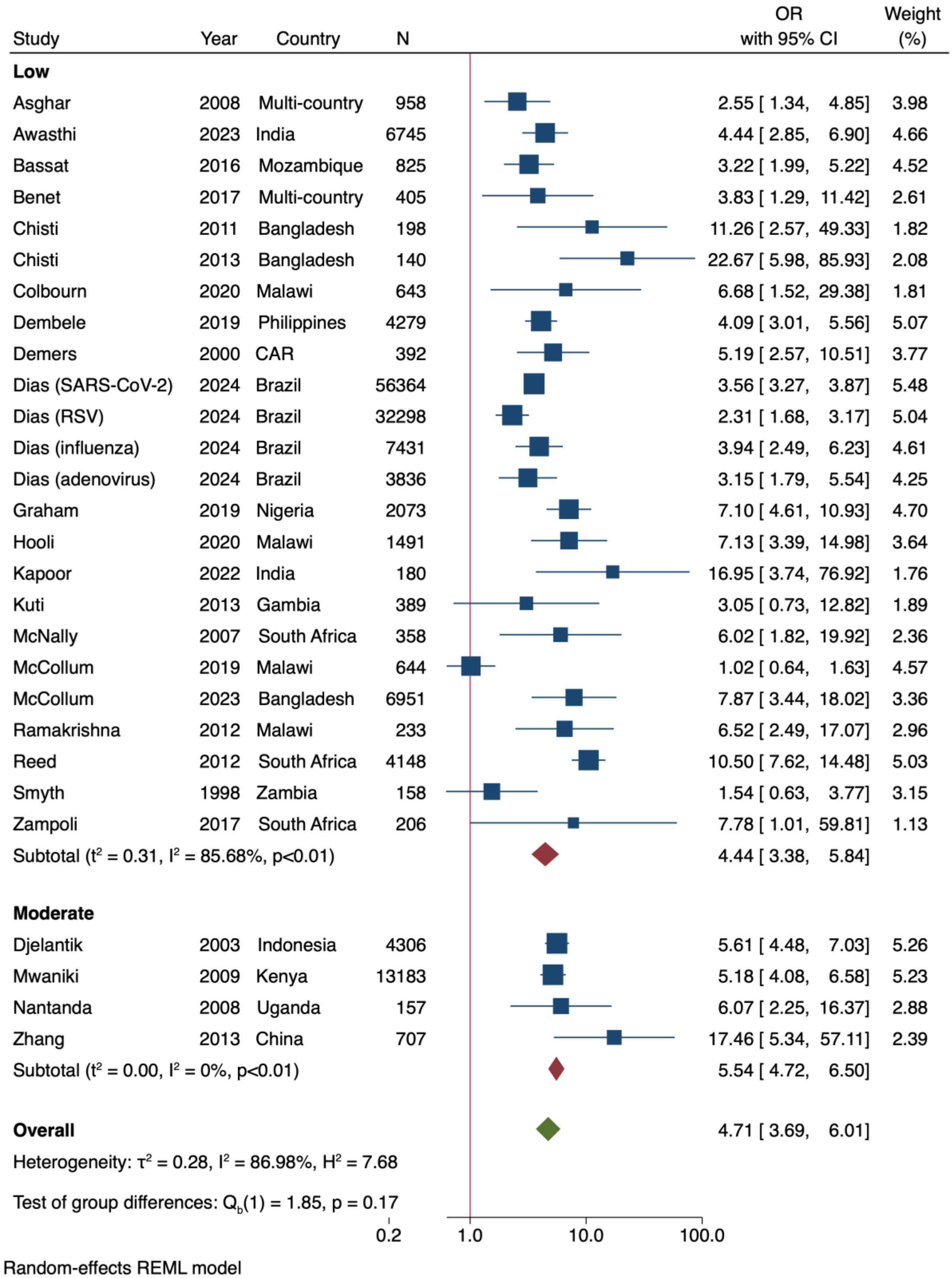

Figure S5: Odds of mortality from lower respiratory infection among children comparing oxygen saturation below versus at or above specified SpO_2 thresholds, using unadjusted odds ratios only.

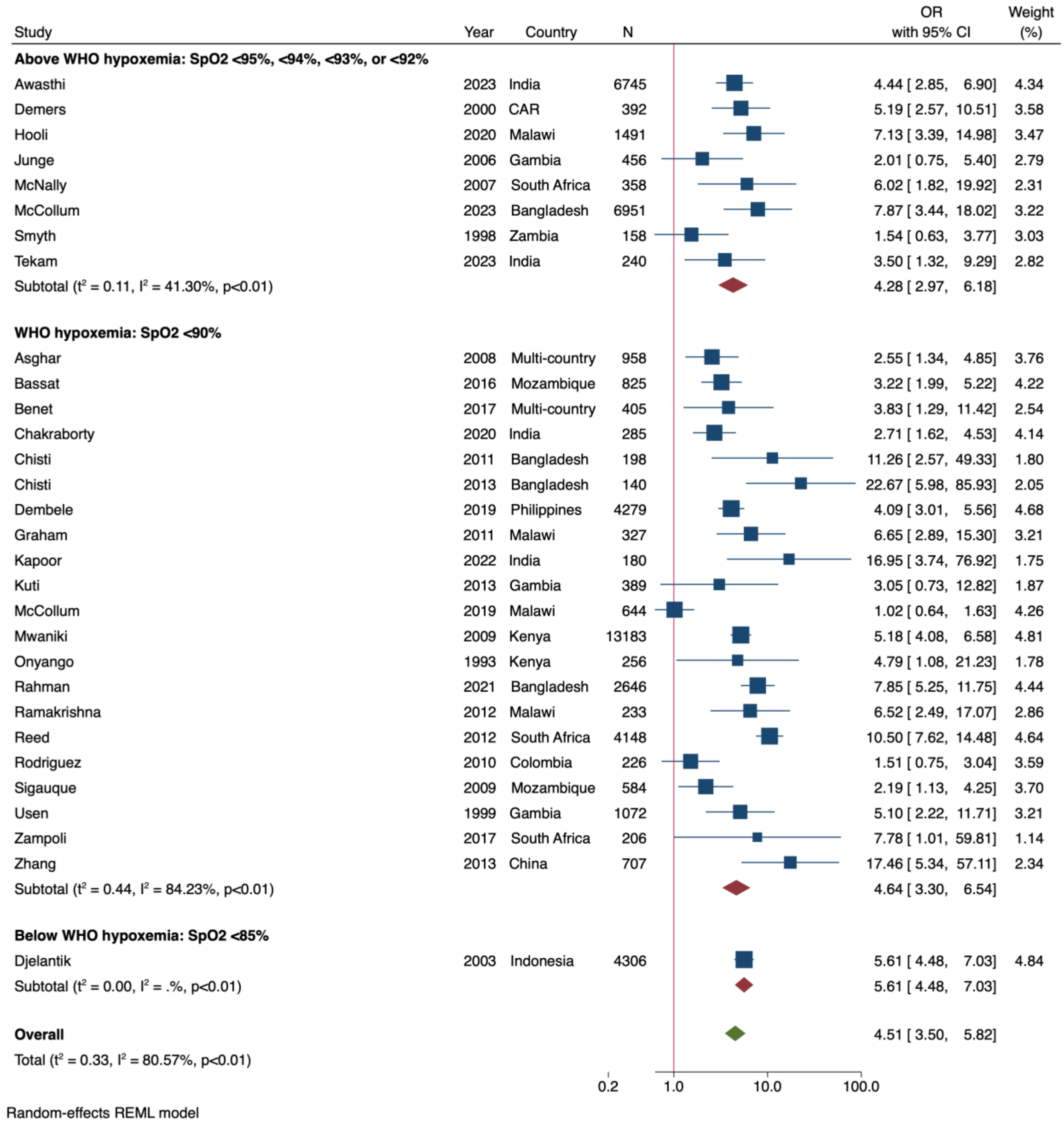

Table S3. Population Attributable Fraction (PAF) for Mortality Associated with Hypoxemia Across Oxygen Saturation Definitions.

**Hypoxemia Category**	**Definition**	**PAF, % (95% CI)**
Above WHO hypoxemia	SpO_2 <95%, <94%, <93%, or <92% vs ≥ respective threshold	46.4% (42.1, 49.8)
WHO hypoxemia	SpO_2 <90% vs ≥90%	48.7% (43.7, 52.3)
Below WHO hypoxemia	SpO_2 <85% vs ≥85%	44.3% (41.9, 46.3)
**Overall**	Hypoxemia of any threshold vs. no hypoxemia	48.1% (44.8, 50.8)
Moderate hypoxemia	SpO_2 90–94% vs 95–100%	12.2% (3.9, 16.7)

PAF = population attributable fraction, CI = confidence interval. PAF estimates represent the proportion of deaths among children with LRI attributable to hypoxemia, assuming a causal relationship.

Table S4. Risk of Bias in the Included Studies

Author, year	Study participation	Study attrition	Prognostic Factor	Outcome measure	Study confounding	Statistical analysis	Overall risk of bias
Abdulkadir, 2015	Low	Low	Low	Moderate	Moderate	Low	Low
Alwadhi, 2017	Low	Low	Low	Moderate	Low	Low	Low
Aroor, 2022	Low	Low	Low	Moderate	High	Moderate	High
Asghar, 2008	Moderate	Low	Low	Low	Low	Low	Low
Awasthi, 2023	Moderate	Low	Low	Low	Low	Low	Low
Bassat, 2016	Moderate	High	Low	Moderate	Low	Low	Low
Benet, 2017	Low	Moderate	Low	Low	Low	Low	Low
Bui-Binh-Bao, 2021	Moderate	Low	Low	Low	Moderate	Moderate	Moderate
Chakraborty, 2020	Moderate	Low	High	Low	Moderate	Moderate	High
Chisti, 2011	Low	Low	Low	Low	Low	Low	Low
Chisti, 2013	Low	Low	Low	Low	Low	Low	Low
Colbourn, 2020	Moderate	Moderate	Low	Low	Low	Low	Low
Dembele, 2019	Low	Moderate	Low	Moderate	Low	Low	Low
Demers, 2000	Low	Low	Low	Low	Moderate	Moderate	Low
Dias, 2024	Low	Low	Moderate	Low	Moderate	Low	Low
Djelantik, 2003	Moderate	Low	Moderate	Low	Low	Moderate	Moderate
Duke, 2001	Low	Low	Low	Low	Moderate	Moderate	Low
Graham, 2011	Moderate	Low	Moderate	Low	High	High	High
Graham, 2019	Low	Unknown	Low	Low	Moderate	Low	Low
Hooli, 2020	Low	Moderate	Low	Low	Low	Low	Low
Junge, 2006	Low	Low	Low	Low	High	High	High
Kapoor, 2022	Low	Low	Low	Low	Low	Low	Low
Kuti, 2013	Low	Low	Low	Low	Moderate	Low	Low
Mc Nally, 2007	Moderate	Low	Low	Low	Low	Moderate	Low
McCollum, 2019	Low	Low	Low	Low	Low	Low	Low
McCollum, 2023	Low	Low	Low	Low	Low	Low	Low
Mwaniki, 2009	Low	Low	Low	Low	Moderate	High	Moderate
Nantanda, 2008	Low	Low	Moderate	Low	Moderate	Moderate	Moderate
Nantanda, 2014	Moderate	Low	Moderate	Low	Low	Low	Low
Odeyemi, 2021	Moderate	Low	Low	Low	Moderate	Low	Low
Onyango, 1993	High	Low	Low	High	High	High	High
Pathak, 2020	High	Low	Moderate	Low	High	High	High
Pranathi, 2023	Low	Low	Low	Low	Low	Moderate	Low
Rahman, 2021	Low	High	Moderate	Moderate	Moderate	Moderate	High
Ramakrishna, 2012	Low	Moderate	Low	Low	Low	Low	Low
Reed, 2012	Low	Low	Moderate	Low	Moderate	Low	Low
Rodriguez, 2010	Unknown	Low	Moderate	Moderate	Moderate	Moderate	High

Shi, 2020	Moderate	Low	Moderate	Low	Moderate	Low	Moderate
Sigauque, 2009	Low	Low	Moderate	Low	High	High	High
Smyth, 1998	Moderate	Low	Low	Low	Low	Moderate	Low
Tekam, 2023	Moderate	Low	Low	Low	Moderate	High	High
Usen, 1999	Moderate	Low	Low	Low	High	High	High
Zampoli, 2017	Low	Low	Low	Low	Low	Low	Low
Zhang, 2013	Low	Low	Moderate	Low	Moderate	Moderate	Moderate

Table S5. Summary of Findings: Association Between Hypoxemia and Mortality

Certainty assessment							Effect	Certainty	Importance
№ of studies	Study design	Risk of bias	Inconsistency	Indirectness	Imprecision	Other considerations	Relative (95% CI)		
33	non-randomised studies	serious[a]	serious[b]	not serious	not serious	publication bias strongly suspected very strong association all plausible residual confounding would reduce the demonstrated effect dose response gradient[c]	**OR 4.36** (3.52 to 5.39)	⨁⨁⨁◯ Moderate[a,b,c]	CRITICAL
5	non-randomised studies	not serious	serious[b,c]	not serious	not serious	publication bias strongly suspected very strong association all plausible residual confounding would reduce the demonstrated effect dose response gradient[c]	**OR 2.27** (1.22 to 4.25)	⨁⨁⨁⨁ High[b,c]	CRITICAL

CI: confidence interval; **OR:** odds ratio

Explanations

a. 8 of 33 studies (24.2%) had a high overall risk of bias using the QUIPS tool.

b. Substantial heterogeneity was observed with variability in effect estimates across studies indicating high heterogeneity.

c. The OR is very large (>2) suggesting a large effect. It is likely that small, negative studies were not published. Confounding may have reduced the demonstrated effect. There is evidence of a dose response gradient
