# Supplementary material for: Hypoxaemia and mortality in children with lower respiratory infection in low-income and middle-income countries: systematic review and meta-analysis
Source: Lancet Glob Health. 2026 Sep 1;14(10):None. doi: 10.1016/j.langlo.2026.104031 (PMC13652795; doi:10.1016/j.langlo.2026.104031)
Supplement: Supplementary file 2 [file mmc2.pdf]

THE LANCET
Global Health

Supplemental appendix 2

This appendix formed part of the original submission and has been peer reviewed. We post it as supplied by the authors.

Supplement to: Hoekstra NE, Joseph KS, Schuh HB, et al. Hypoxaemia and mortality in children with lower respiratory infection in low-income and middle-income countries: systematic review and meta-analysis. *Lancet Glob Health* 2026. https://doi.org/10.1016/j.langlo.2026.104031

Equitable Partnership Declaration

If any questions do not apply to your study, please indicate "N/A" for "not applicable. For more information on how to complete this form see the Information for Authors document.

Researcher considerations

1. Please detail the involvement that researchers who are based in the country or countries of study had during a) study design; b) clinical study processes, such as processing blood samples, prescribing medication, or patient recruitment; c) data interpretation; and d) manuscript preparation, commenting on all aspects. If they were not involved in any of these aspects, please explain why.

This should include a thorough description of their leadership roles in the study. Are local researchers named in the author list or the acknowledgements, or are they not mentioned at all (and, if not, why)? Please also describe the involvement of early career researchers based in the location of the study. Some of this information might be repeated from the Contributors section in the manuscript. Note: we adhere to ICMJE authorship criteria for naming authors on a paper.

**a) Study design:**<br>This study is a systematic review and meta-analysis of published studies conducted across multiple low- and middle-income countries (LMICs), rather than a primary study conducted in a single country or set of countries. Study design was led by a multidisciplinary group of investigators, including multiple authors with expertise in pediatric respiratory research and direct experience working in LMIC settings, including South Africa, Malawi, Bangladesh, and Nigeria. Investigators based in LMICs, including Dr. Eric McCollum and Dr. Heather Zar, contributed to study conception, design, and framing of clinically relevant research questions.
**b) Clinical study processes:**<br>Not applicable. This study did not involve primary data collection or clinical procedures, such as patient recruitment, treatment administration, or laboratory processing. All data were extracted from previously published studies.
**c) Data interpretation:**<br>Data interpretation was conducted collaboratively by the full author group, including investigators based in LMICs with extensive clinical and research experience in pediatric LRI care in resource-constrained settings. These investigators provided critical contextualization of findings, particularly with respect to clinical relevance, health-system constraints, and interpretation of hypoxemia thresholds in LMIC settings.
**d) Manuscript preparation:**<br>Manuscript preparation was led by NEH and KSJ, with substantial input and iterative revisions from all co-authors. Authors based in LMICs, including those working in

South Africa, Malawi, Bangladesh, and Nigeria, contributed to manuscript review, interpretation of results, and framing of policy-relevant implications. All authors meet ICMJE authorship criteria and are listed on the author list.

2. How was funding used to remunerate and enhance the skills of researchers in the countries of study? And how was funding used to improve research infrastructure at the study sites?

   Potentially effective investments into long-term skills and opportunities within local institutions could include training or mentorship in analytical techniques and manuscript writing, opportunities to lead all or specific aspects of the study, financial remuneration rather than requiring volunteers, and other professional development and educational opportunities.

   Improvements to research infrastructure could include funding extended trial designs (eg, platform trials), establishment of long-term contracts for research staff, building research facilities, and setting up local control of funding allocation.

Skills:
No funding was received to support this study. As a result, there were no dedicated training or capacity-building activities tied to this project. However, the study involved collaboration across institutions in high-income and LMIC settings, with shared leadership in study design, data interpretation, and manuscript development, providing opportunities for collaborative scientific exchange.

Research infrastructure:
No funding was used to support research infrastructure, as this work was based on analysis of existing published data and did not involve new data collection or development of physical infrastructure.

3. How did you safeguard the researchers who implemented the study?

   Please describe how you guaranteed safe working conditions for study staff, including provision of appropriate personal protective equipment, protection from violence, and prevention of overworking.

Not applicable. This study did not involve fieldwork, clinical activities, or direct engagement with study participants or communities. All work was conducted through analysis of published data and collaborative manuscript preparation.

Benefits to the communities and regions of study

4. How does the study address the research and policy priorities of its location?

How were the local priorities determined and then used to inform the research question? Who decided which priorities to take forward? Which elements of the study address those priorities?

This study addresses mortality from lower respiratory infections, a major global child health priority in LMIC settings where the burden is highest. The research question was informed by longstanding gaps in evidence related to risk stratification and clinical thresholds for hypoxemia, which are central to WHO management guidelines and frontline clinical decision-making in LMIC health systems. The study directly evaluates questions relevant to triage, referral, and oxygen use in low-resource settings.

5. How will research products be shared in the community of study?

For instance, will you be providing written or oral layperson summaries for non-academic information sharing? Will study data be made available to institutions in the region(s) of study? The Lancet Group *encourages authors to translate the summary (abstract) into relevant languages after paper editing; do you intend to translate your summary?*

Findings will be disseminated through publication in an open-access journal to ensure accessibility to researchers, clinicians, and policymakers globally, including in LMIC settings. Data used in this study are derived from published sources and are publicly available. All search strategies and study-level characteristics are reported in our manuscript or supplemental files. Additional extracted data and analytic files will be available upon request. We would support translation of the abstract into relevant languages if requested by the journal.

6. How were individuals, communities, and environments protected from harm?
This study used only previously published, aggregate data and did not involve individual-level data. As such, there was no risk to patient privacy, confidentiality, or potential stigma.

a) *How did you ensure that sensitive patient data were handled safely and respectfully? Was there any potential for stigma or discrimination against participants arising from any of the procedures or outcomes of the study?*

This study used only previously published, aggregate data and did not involve individual-level data. As such, there was no risk to patient privacy, confidentiality, or potential stigma.

b) *Might any of the tests be experienced as invasive or culturally insensitive?*

Not applicable. This study did not involve direct participant interaction, biological sampling, environmental interventions, infrastructure development, or use of healthcare resources.

c) *How did you determine that work was sensitive to traditions, restrictions, and considerations of all cultural and religious groups in the study population?*

Not applicable. This study did not involve direct participant interaction, biological sampling, environmental interventions, infrastructure development, or use of healthcare resources.

d) *Were biowaste and radioactive waste disposed of in accordance with local laws?*

Not applicable. This study did not involve direct participant interaction, biological sampling, environmental interventions, infrastructure development, or use of healthcare resources.

e) *Were any structures built that would have impacted members of the community or the environment (such as handwashing facilities in a public space)? If so, how did you ensure that you had appropriate community buy-in?*

Not applicable. This study did not involve direct participant interaction, biological sampling, environmental interventions, infrastructure development, or use of healthcare resources.

f) *How might the study have impacted existing health-care resources (such as staff workloads, use of equipment that is typically employed elsewhere, or reallocation of public funds)?*

Not applicable. This study did not involve direct participant interaction, biological sampling, environmental interventions, infrastructure development, or use of healthcare resources.

7. Confirm that local ethics review was sought, and please provide the approval number. If not sought, please explain why.

Not applicable. This study is a systematic review and meta-analysis of published data and did not involve human participants or require ethical approval.

Secondary analyses

8. Have the data analysed in your study been extracted from another source, such as a national survey, rather than being directly collected by the authors of this paper?

YES / No / N/A

If the authors of this paper were not involved in data collection, how were the findings interpreted with sufficient contextual knowledge?
The Lancet Group *believe contextual understanding is crucial for informed data analysis and interpretation*.

The study is a systematic review and meta-analysis based on previously published studies. Interpretation was conducted by a multidisciplinary research team with substantial clinical and research experience in LMIC settings, ensuring appropriate contextual understanding of the findings.

---

9. Please provide the title (eg, Dr/Prof, Mr/Mrs/Ms/Mx), name, and email address of an author who can be contacted about this statement.

**Name:** Dr. Nadia E. Hoekstra, MD<br>**Email:** Nadia_Hoekstra@med.unc.edu

10. Finally, please provide the title and name of an author from one country of study who has seen and approved this form.

**Name:** Dr. Eric D. McCollum, MD, MPH, Associate Professor
